# Shank and Zinc Mediate an AMPA Receptor Subunit Switch in Developing Neurons

**DOI:** 10.3389/fnmol.2018.00405

**Published:** 2018-11-09

**Authors:** Huong T. T. Ha, Sergio Leal-Ortiz, Kriti Lalwani, Shigeki Kiyonaka, Itaru Hamachi, Shreesh P. Mysore, Johanna M. Montgomery, Craig C. Garner, John R. Huguenard, Sally A. Kim

**Affiliations:** ^1^Department of Neurology & Neurological Sciences, School of Medicine, Stanford University, Stanford, CA, United States; ^2^Neurosciences Graduate Program, School of Medicine, Stanford University, Stanford, CA, United States; ^3^Department of Material Science & Engineering, School of Engineering, Stanford University, Stanford, CA, United States; ^4^Department of Synthetic Chemistry & Biological Chemistry, Graduate School of Engineering, Kyoto University, Kyoto, Japan; ^5^Department of Psychological & Brain Sciences, Johns Hopkins University, Baltimore, MD, United States; ^6^Department of Physiology and Centre for Brain Research, University of Auckland, Auckland, New Zealand; ^7^German Center for Neurodegenerative Diseases (DZNE), Charité—Universitätsmedizin Berlin, Berlin, Germany

**Keywords:** zinc signaling, AMPA receptor (AMPAR), GluA2 (GluR2), GluA1 (GluR1), Shank2, Shank3, synaptic development, subunit switch

## Abstract

During development, pyramidal neurons undergo dynamic regulation of AMPA receptor (AMPAR) subunit composition and density to help drive synaptic plasticity and maturation. These normal developmental changes in AMPARs are particularly vulnerable to risk factors for Autism Spectrum Disorders (ASDs), which include loss or mutations of synaptic proteins and environmental insults, such as dietary zinc deficiency. Here, we show how Shank2 and Shank3 mediate a zinc-dependent regulation of AMPAR function and subunit switch from GluA2-lacking to GluA2-containing AMPARs. Over development, we found a concomitant increase in Shank2 and Shank3 with GluA2 at synapses, implicating these molecules as potential players in AMPAR maturation. Since Shank activation and function require zinc, we next studied whether neuronal activity regulated postsynaptic zinc at glutamatergic synapses. Zinc was found to increase transiently and reversibly with neuronal depolarization at synapses, which could affect Shank and AMPAR localization and activity. Elevated zinc induced multiple functional changes in AMPAR, indicative of a subunit switch. Specifically, zinc lengthened the decay time of AMPAR-mediated synaptic currents and reduced their inward rectification in young hippocampal neurons. Mechanistically, both Shank2 and Shank3 were necessary for the zinc-sensitive enhancement of AMPAR-mediated synaptic transmission and act in concert to promote removal of GluA1 while enhancing recruitment of GluA2 at pre-existing Shank puncta. These findings highlight a cooperative local dynamic regulation of AMPAR subunit switch controlled by zinc signaling through Shank2 and Shank3 to shape the biophysical properties of developing glutamatergic synapses. Given the zinc sensitivity of young neurons and its dependence on Shank2 and Shank3, genetic mutations and/or environmental insults during early development could impair synaptic maturation and circuit formation that underlie ASD etiology.

## Introduction

Autism Spectrum Disorders (ASDs) have symptom onset during the first three years of life, a period characterized by intense formation and refinement of synaptic connections. Therefore, it is not surprising that many ASD-associated genes encode synaptic proteins, such as neuroligins (NL3 and NL4; Jamain et al., 2003; Laumonnier et al., 2004), neurexins (Nrx1; Kim et al., 2008), and the Shank family of proteins (Durand et al., 2007; Berkel et al., 2010; Leblond et al., 2012; Sato et al., 2012). This suggests that changes in synaptic structure and function are causally associated with ASDs (Chen et al., 2014; Bourgeron, 2015). Synaptic deficits are also strongly linked with ASD-related environmental insults (such as prenatal inflammation and zinc deficiency) during the critical periods of brain development (Yasuda et al., 2011; Forrest et al., 2012; Grabrucker et al., 2014; Giovanoli et al., 2016), underscoring synapses as a potential focus for genetic and environmental interactions. Therefore, a better understanding of signaling pathways regulating synapse development is essential to discover effective pharmacotherapies for ASDs.

Mutations in the human *SHANK2* and *SHANK3* genes have been implicated in ASDs. Disruptions of these molecules serve as key models to study the underlying neuronal and synaptic dysfunction of ASDs (Harony et al., 2013; Jiang and Ehlers, 2013). The family of Shank proteins (Shank1, Shank2 and Shank3) are key multidomain molecules at excitatory synapses that interact with multiple glutamatergic receptors, cell adhesion molecules and cytoskeletal proteins (Boeckers et al., 1999; Naisbitt et al., 1999; Sheng and Kim, 2000; Arons et al., 2012). While they share high structural similarity and all localize to postsynaptic sites, Shank proteins are differentially expressed during development with Shank2 and Shank3 peaking before Shank1 (Boeckers et al., 1999; Grabrucker et al., 2011). Thus, Shank2 and Shank3 are thought to be critical for plastic aspects of synaptic activity during development and are the focus of this work. For example, Shank2 is thought to play a key role in growth cone function and nascent synapse formation (Du et al., 1998; Bresler et al., 2004) and Shank3 for dendritic spine morphogenesis (Roussignol et al., 2005). Both Shank2 and Shank3 knockout mice show impairments of synaptic plasticity and learning (Bozdagi et al., 2010; Peça et al., 2011; Won et al., 2012; Lee E. J. et al., 2015). Together, these data support the important roles of Shank2 and Shank3 in synapse formation and plasticity.

Shank2 and Shank3 have also been implicated in the maturation of synapses. For example, loss of Shank2 in mice resulted in reduced GluA1 levels, delayed synaptic maturation, and a reduction of AMPA receptor (AMPAR) function (Peter et al., 2016; Wegener et al., 2017). Similarly, Shank3 was shown to play a key role in the postnatal development of excitatory synapses, such that loss of Shank3 increased the AMPA/NMDA ratio and impaired the developmental AMPAR subunit switch (Peça et al., 2011; Bariselli et al., 2016). Such cellular and molecular deficits and autistic-like phenotypes in these mouse models were rescued by both genetic and pharmacological restoration of AMPAR functions (Bariselli et al., 2016; Mei et al., 2016). These findings suggest that Shank2 and Shank3 are critical for AMPAR recruitment and functionality in multiple brain circuits. Mechanistically, these Shank proteins could induce such changes by direct modulation of GluA1 trafficking via the Rich2 or mGluR dependent pathways, and/or indirectly through an interaction with GluA2 via GRIP (Sheng and Kim, 2000; Uchino et al., 2006; Verpelli et al., 2011; Raynaud et al., 2013). Understanding the interactions between Shank proteins and AMPAR will provide key insights into how they operate to regulate subunit switching of AMPARs during development.

Zinc has been shown to regulate the structure and function of Shank2 and Shank3 through its binding to the C-terminal sterile alpha motif (SAM) domain in these proteins. Both of these proteins require zinc binding for their synaptic localization, oligomerization, mobility and trans-synaptic signaling (Boeckers et al., 2005; Baron et al., 2006; Grabrucker et al., 2011; Arons et al., 2016). ASD-associated mutations in the SAM domain are associated with severe synaptic deficits in cell culture and mouse models (Baron et al., 2006; Durand et al., 2012; Speed et al., 2015). Interestingly, prenatal zinc deficiency also reduced expression level of Shank2 and Shank3 as well as Shank-binding partners, such as GluA1, suggesting that these Shank proteins act as mediators of zinc effects on synaptic function (Grabrucker et al., 2014). Furthermore, Shank3 was shown to be necessary for zinc-sensitive potentiation of AMPAR evoked EPSCs in young hippocampal neurons (Arons et al., 2016). In most neurons, basal free intracellular zinc is tightly regulated at very low picomolar concentrations (Maret, 2017). One endogenous source of free zinc for Shank modulation includes zinc release from synaptic vesicles which can enter postsynaptic sites via different ion channels, such as calcium-permeable AMPAR, NMDAR and voltage-gated calcium channels (Vogt et al., 2000; Frederickson et al., 2006; Vergnano et al., 2014). Synaptic activity could also trigger zinc release from postsynaptic zinc buffers, such as Metallothionein III or mitochondria (Masters et al., 1994; Cuajungco and Lees, 1998; Cole et al., 2000; Lee et al., 2003; Bossy-Wetzel et al., 2004). We thus hypothesize that zinc is well positioned to serve as a dynamic regulator to activate Shank-dependent pathways, such as regulating AMPAR subunit composition during synaptic development.

*Here, we examine how Shank2, Shank3 and zinc mechanistically regulate AMPAR subunit composition and function in developing synapses*. Our data reveal that during development, these Shank proteins exhibit increased synaptic localization in parallel with GluA2-containing AMPAR, supporting the hypothesis that they might play a role in the maturation of AMPARs. To test this, we studied whether neuronal activity regulated the level of postsynaptic zinc at glutamatergic synapses and in turn affected Shank and AMPAR localization and activity. We showed that K^+^-induced neuronal depolarization elevated postsynaptic zinc transiently and reversibly. Zinc elevation was found to enhance synaptic efficacy by recruiting GluA2 and dispersing GluA1 at Shank-containing synapses in young neurons. Importantly, knockdown of either Shank2 or Shank3 function abolished the zinc-induced enhancement of AMPAR-mediated transmission, indicating that Shank2 and Shank3 are critical mediators of a zinc-dependent AMPAR signaling pathway. Together, these data provide a potential mechanistic link between genetic mutations in Shank proteins and zinc deficiency in the etiology of ASD.

## Materials and Methods

### Reagents

#### Drugs

Picrotoxin, 2,3-dihydroxy-6-nitro-7-sulfamoyl-benzo(f)quinoxa-line (NBQX), 6-cyano-7-nitroquinoxaline-2,3-dione (CNQX) and N-(2,6-dimethylphenylcarbamoylmethyl)triethylammonium bromide (QX314) were purchased from Tocris (R&D System Inc., Minneapolis, MN, USA). D-2-amino-5-phosphonopentanoic acid (D-AP5) and tetrodotoxin (TTX) were obtained from Abcam, Inc.

#### Antibodies

Primary antibodies used for immunocytochemistry and/or western blots included: Homer1 (1:750, Synaptic Systems; 160003), Shank2 (1:1,000, Synaptic Systems; 162204), Shank3 (1:500, Synaptic Systems; 162302 and 162304), GluA1 and GluA2 [1:8 for surface staining and 1:100 for whole cell staining, Millipore; PC246 and MAB397], VGluT1 (1:100, NeuroMab; N28/9), Shank2 (1:100, Neuromab; N23B/6), Shank3 (1:100, NeuroMab; N367/62), PSD-95 (1:100, NeuroMab; K28/43), MAP2 (1:5,000, Abcam; ab5392), green fluorescent protein (1:1,000, Abcam; ab13970), actin (1:1,000, Abcam; ab8227), and Shank2 (1:250, Cell Signaling; 12218). A custom-made VGluT1 antibody (1:500, polyclonal rabbit) was generously provided by Dr. Richard Reimer (Stanford University). All secondary antibodies (1:500, A11029, A11034, A11036, A11039, A11041, A11075, A21235 and A21449) were obtained from Life Technologies with the exception of the Dylight-350 antibody (1:250, Thermo Fisher; SA5-10069) and the HRP-conjugated antibodies (1:10,000, rabbit, mouse or guinea pig; Jackson ImmunoResearch; 706-035-148, 115-035-003 and 111-035-144).

#### Reagents and Chemicals

Cell culture reagents were purchased from Life Technologies [Trypsin-EDTA (0.05%), TrypLE, N-2-hydroxyethylpiperazine-N-2-ethane sulfonic acid (HEPES), Anti-Anti, B-27 supplement along with Neurobasal, Dulbecco’s modified Eagle’s media (DMEM) and Minimum Essential Media (MEM) medias], Sigma-Aldrich [Cytosine β-D-arabinofuranoside (Ara-C), poly-D-lysine (70–150 kDa), Hank’s Balanced Salt Solution (HBSS), 1,4-Piperazinediethanesulfonic acid (PIPES), 3-(N-morpholino)propanesulfonic acid (MOPS), Ethylene glycol-bis(2-aminoethylether)-*N,N,N′,N′*-tetraacetic acid (EGTA), insulin, N-Acetyl Cysteine (NAC), hydrocortisone, sodium pyruvate and GlutaMAX], Worthington (Trypsin, Papain, Papain Dissociation Kit, and DNase), Atlanta Biologicals [Horse Serum (HS) and Fetal Bovine Serum (FBS)]. Zinc indicators (FluoZin-3 AM and Newport Green DCF) were obtained from Life Technologies. Vitamin MEM solution, amino acid MEM solution, ZnCl_2_ (0.1 M stock solution), *N,N,N′,N′*-Tetrakis (2-pyridylmethyl)ethylenediamine (TPEN), 2-Mercaptopyridine *N*-oxide sodium salt (pyrithione) were all purchased from Sigma-Aldrich. For GluA2 surface labeling, Alexa 488-CAM2 and Alexa 647-CAM2 were designed and synthesized by the Hamachi research group (Kyoto University; Wakayama et al., 2017).

### DNA Constructs

The mApple expression plasmid was generously provided by Dr. Neal Waxham (University of Texas Health Science Center at Houston).

### Short-Hairpin RNA (shRNA) Design and Cloning

Sequences of *Rattus norvegicus* Shank2 and Shank3 from GenBank (NIH) were utilized to design short-hairpin RNA (shRNA) specifically targeting either Shank2 or Shank3. Online software (sidirect2.rnai.jp) was utilized to identify candidate sequences using criteria from three references (Amarzguioui and Prydz, 2004; Reynolds et al., 2004; Ui-Tei et al., 2004) with a maximal melting temperature (T_m_) of 21°C. Literature searches for shRNA targeting Shank2 or Shank3 were also performed to provide a secondary selection of our custom designed shRNA. All candidate sequences were then further examined and modified using the iRNAi software (mekentosj.com). Forward and reverse oligo sequences were synthesized, annealed and cloned into the pZoff vector (see below; Leal-Ortiz et al., 2008). The different elements included: recognition sites of restriction enzymes used for cloning (underlined), structural elements of shRNA (5′ or 3′ Prefix—**Bold**, Loop –**Bold, underlined**) and specific shRNA sequences (red; Supplementary Table S1).

**Forward**: GATC**CCC** shRNA seq **TTCAAGAGA**
complement shRNA seq **TTTTTGGAAA**
**Reverse**: AGCTT**TTCCAAAAA** shRNA seq **TCTCTTGAA**
complement shRNA seq **GGG**

Constructs were transfected into rat hippocampal neurons, immunostained for both Shank2 and Shank3 and assessed for knockdown efficiency at synapses (Supplementary Tables S1, S2). Based on these data, we employed the following nucleotide siRNA sequences that target rat and mouse Shank2 (5′-3′: GGATAAACCGGAAGAGATA from *Rattus norvegicus* Shank2, GenBank accession no. NM133441.1; Berkel et al., 2012) or Shank3 (5′-3′: GTTTGGAGTCTGGACTAAG, GenBank accession no. NM021676.1; Bidinosti et al., 2016). As a control, a luciferase-targeting shRNA sequence was used (5′-3′: CTTACGCTGAGTACTTCGA; Bidinosti et al., 2016). For lentivirus production, the H1 promoter and shRNA elements were subcloned into a pFUGW H1 vector.

### Cell Culture

#### Human Embryonic Kidney 293T Cells

HEK293T (ATCC CRL-3216) were maintained in DMEM (Invitrogen) supplemented with 10% FBS in a humidified 5% CO_2_ incubator at 37°C and passaged every 2–3 days using TrypLE and mechanical trituration. Cells used for viral production were passaged fewer than 10 times after their initial acquisition from ATCC.

#### Primary Rat Hippocampal Neurons

Primary rat hippocampal neurons were prepared according to a Banker culture protocol from hippocampi of wildtype Sprague-Dawley rat embryos (embryonic day 18 or 19) with mixed gender (Kaech and Banker, 2006). Rats were handled in accordance with Stanford University Administrative Panel on Laboratory Animal (APLAC) guidelines (Protocol #14607). Hippocampi were dissected in ice cold HBSS supplemented with 10 mM HEPES pH 7.4, glucose and Anti-Anti and digested in trypsin in Neurobasal media at 37°C for 15 min. Cells were dissociated and plated at a density of 178 cells per mm^2^ on poly-D-lysine-coated coverslips (Carolina Biological Supply Company) with paraffin feet in warmed neuronal plating media (0.6% glucose, 10% HS and 100 μM sodium pyruvate in MEM). After 1 h, coverslips were transferred in pairs to 60-mm dishes containing a glial feeder layer, where they were inverted and maintained in Neurobasal media containing B-27 and GlutaMAX in a humidified 5% CO_2_ incubator at 37°C. Neurons were fed with a half media exchange twice per week. At 7 days *in vitro* (DIV 7), cells were treated with 800 nM Ara-C for approximately 24 h.

#### Primary Astrocyte Cultures

Primary astrocytes from the cortex of P0–2 rats with mixed gender were prepared according to the Papain Dissociation Kit. In brief, cortices from neonate rats were dissected, placed in dissection media, chopped into small pieces and then digested with papain and DNase. The astrocytes were cultured in glial media (10% FBS, 100 μM sodium pyruvate, 5 μg/ml N-acetyl-L-cysteine, 5 μg/ml insulin and 5 ng/ml hydrocortisone in DMEM). Microglia and other contaminating cells were shaken off of a confluent monolayer at one week, after which cells were allowed to recover. Upon reaching 80%–90% confluence, astrocytes were passaged, plated or harvested for cryopreservation. For use in Banker cultures, astrocytes were passed and plated one day prior to neuron preparation.

### Gene Delivery

#### Neuronal Transfections

Neuronal transfections were performed using Lipofectamine 2000 as described by the manufacturer (Invitrogen) with the following modifications. Neurons were transfected at DIV 9–10 with a 1.8:1 ratio of Lipofectamine to DNA in transfection media (Neurobasal supplemented with Glutamax). After incubation at 37°C for 80 min, coverslips were placed back into their original 60 mm dishes with a half media exchange. Hippocampal neurons were incubated for 2–4 days prior to imaging experiments.

#### Lentivirus Production

To create lentivirus for expression of a given protein or shRNA, HEK293T cells were transfected in suspension with the lentiviral transfer plasmids FU-X-Wm, envelope plasmid pCMV-VSV-G, and packaging plasmid SPAX2 (2.8:1:1.5 ratio, respectively) using Lipofectamine 2000 to generate replication-incompetent lentivirus. The transfection media was replaced with complete Neurobasal media 6 h post-transfection, and cells were moved to a humidified 5% CO_2_ incubator at 32°C. After 48–52 h, the lentivirus containing supernatant was collected, passed through a 0.45 μm filter to remove cellular debris and stored at -80°C until use. Biological Safety Level 2+ (BSL-2+) guidelines were applied for all lentiviral production and handling.

#### Lentiviral Infection

Hippocampal cultures were infected by the addition of 100–130 μL of lentivirus, to hippocampal neurons in 60 mm dishes at DIV 0–5 to infect 80%–100% of neurons depending on the day of infection.

### Electrophysiological Recordings

#### General Experimental Conditions

Neurons were transferred to a recording chamber and perfused at a rate of 0.5 ml/min with HibernateE [in mM: 81.4 NaCl, 1.8 CaCl_2_, 0.0025 Fe(NO_3_)_3_*9H_2_O, 5.26 KCl, 0.812 MgCl_2_*6H_2_O, 0.880 NaHCO_3_, 0.906 NaH_2_PO_4_, 34 D-glucose, 10 MOPS, 0.227 sodium pyruvate, Vitamin MEM (1:27) and Amino Acid MEM (1:26), pH 7.3, 235–240 mOs] at RT. Hibernate mimics the composition of Neurobasal media and improves both the stability and duration of recordings. Neurons were visualized using a 60× 0.9 NA LUMPlanFl/IR objective (Olympus Corporation) using differential contrast optics on an Axioskop 50 FS microscope (Zeiss) equipped with an X-Cite 120Q excitation light source. Pyramidal neurons were selected for electrophysiological recordings based on their pyramidal or pear-shaped somata with 3-4 primary dendrites.

#### Whole Cell Patch Clamp Recordings of AMPAR-Mediated Miniature EPSCs

Whole-cell recordings in voltage-clamp mode were obtained using borosilicate glass electrodes (Sutter Instrument) with a tip resistance of 3–7 MΩ. The internal solution contained (in mM): 114.5 gluconic acid, 114.5 CsOH, 2 NaCl, 8 CsCl, 10 MOPS, 4 EGTA, 4 MgATP and 0.3 Na_2_GTP, pH 7.3, adjusted with CsOH.

Signals were amplified with a Multiclamp 700A amplifier, sampled at 20 kHz, filtered at 2.4 kHz, acquired using a Digidata 1440A digitizer and pClamp 10 (all from Molecular Devices). Cells were held at -60 mV, and AMPAR-mediated miniature EPSCS were isolated using bath application of TTX (1 μM), D-AP5 (50 μM) and picrotoxin (100 μM).

ZnCl_2_ (10 μM) was bath applied in HibernateE to the cultured neurons. Series resistance (R_s_) was monitored throughout the duration of all recordings, and data were excluded if R_s_ increased >20%.

#### Evoked AMPAR EPSC Recordings

AMPAR-mediated evoked EPSCs were pharmacologically isolated by bath application of a NMDAR blocker (50 μM D-AP5) and GABAR blocker (100 μM picrotoxin). For these recordings, the internal pipette solution contained (in mM) 101 gluconic acid, 101 CsOH, 11 KCl, 10 MOPS, 2.9 QX 314, 1 CaCl_2_, 5 EGTA, 2 MgATP, 0.3 Na_2_GTP and 50 μM spermine, pH 7.3 adjusted with CsOH (250 mOsm). In addition to D-AP5 and picrotoxin, a very low concentration of NBQX (0.05 μM) was added to the bath to reduce spontaneous AMPAR-mediated synaptic activity that occurred under conditions of disinhibition (Kumar et al., 2002). To stimulate evoked AMPA EPSCs, a platinum parallel bipolar electrode (FHC) was placed in close proximity (~1.5 mm) to the recorded neurons. Synaptic activity was evoked by delivering current pulses of 4–5 mA for 0.5 ms at intervals of 20 s. Post data collection, membrane potentials were corrected for a liquid junction potential of 18 mV.

#### Analysis

We detected and analyzed miniature EPSCs with wDetecta, a custom postsynaptic current detection program^1^. Numerical values are given as median ± SEM unless stated otherwise. Quantification of zinc conditions was performed using data after 10 min of zinc application to capture the plateau phase of the zinc effects. Wilcoxon matched-pairs signed rank tests were applied to compare between baseline and ZnCl_2_ conditions for young or mature neurons. For shRNA experiments, the effect of genotypes on the zinc response were tested by two-way analysis of variance (ANOVA) and Sidak correction multiple *post hoc* comparison was applied to determine the difference between baseline and ZnCl_2_ within the same genotype. Statistical significance was set at *p* < 0.05.

For cumulative probability distributions, each cohort population was composed of a random selection of equal number of events per condition from each cell (i.e., 600 events from 12 cells). Kolmogorov-Smirnov (K.S.) test was applied to determine statistical significance (*p* < 0.005) for experiments with two samples. Kruskal-Wallis one-way ANOVA with Dunn’s correction *post hoc* multiple comparison was used for experiments with more than two samples.

For evoked AMPAR EPSC, current response at each holding voltage was measured by averaging the value within 3–6 ms of peak current using pClamp 10 (Molecular Devices). The I/V slope for negative and positive responses was calculated separately for each condition (baseline or ZnCl_2_) of each cell using linear regression in Excel 2016 (Microsoft Office). For the population I/V curve, current responses were normalized to the value at the most negative potential. The rectification index was defined as the ratio of the I/V slope of negative responses over that of positive responses. The correlation between the rectification index and change in RI induced by ZnCl_2_ application was calculated using a two-tailed Pearson correlation coefficient analysis, and statistical significance was set at *p* < 0.05.

All graphs and statistical analyses were done in Prism 6.0 (GraphPad Software).

### Immunoblot Analysis

Immunoblots of cellular lysates were prepared from lentiviral infected hippocampal neurons as described previously (Hsieh et al., 2016; Okerlund et al., 2017). In brief, neurons were infected with a lentiviral vector expressing shRNA and enhanced green fluorescent protein (eGFP) on DIV 1. Lysates from these neurons were harvested at DIV 14–15 in Laemmli loading buffer (Bio-Rad) with β-mercaptoethanol (Sigma). Lysates were loaded on either a 4%–15% or 4%–20% polyacrylamide gels (Bio-Rad) and transferred to a polyvinylidene fluoride (PVDF) membrane (Bio-Rad). After washing (0.1% Triton X-100 in phosphate buffered saline (PBS)) and blocking (5% non-fat powdered milk and 0.1% Triton X-100 in PBS) overnight at 4°C, membranes were probed with primary and secondary antibodies in blocking solution. Protein bands were visualized by West Dura ECL reagents (GE Healthcare). Membranes were either blotted simultanously with Shank and actin antibodies or blotted with Shank antibodies first, then stripped using Restore Western Blot Stripping Buffer (Thermo Fisher) and blotted again for actin to standardize protein levels. Representative blots of relevant protein bands are shown in the figures, and full blots are provided in the Supplementary Material.

### Immunocytochemistry

#### Whole-Cell Staining

For whole-cell staining, wild-type (WT) or transfected rat hippocampal neurons were washed at room temperature (RT) with HibernateE solution. Cells were then placed in fixative solution [in mM: 60 PIPES, 25 HEPES, 120 sucrose, 2 MgCl_2_, 10 EGTA and 4% paraformaldehyde (PFA), pH 7.4] for 10 min at RT. After fixation, cells were washed twice with PBS, permeabilized with 0.25% Triton X-100 (Thermo Scientific) in PBS for 2 min and washed twice with PBS at RT. Fixed and permeabilized cells were incubated in blocking solution (2% glycine, 2% bovine serum albumin, 0.2% gelatin, and 50 mM NH_4_Cl in PBS, pH 7.4) for 30 min at RT, and then primary antibodies were applied in blocking solution overnight at 4°C. After the primary antibody incubation, cells were washed three times with blocking solution, followed by the addition of secondary antibodies for 1–2 h at RT. After secondary antibody application, cells were washed three times with PBS and rinsed quickly with water. Neurons were then mounted with Fluoromount-G (Southern Biotech) on pre-cleaned glass slides.

#### Surface Staining of AMPAR Subunits

The protocol for surface staining of AMPAR subunits was modified from published studies (Lu et al., 2001; Mangiavacchi and Wolf, 2004; Park et al., 2004). Coverslips were incubated in HibernateE solution with or without 10 μM ZnCl_2_ for 20 min at RT. After treatment, coverslips were incubated with GluA1 or GluA2 antibodies in HibernateE with 2% BSA at 4°C on ice for 1 h. After primary antibody labeling, coverslips were washed twice with 2% BSA in HibernateE and placed in fixative solution. Neurons were then permeabilized and co-stained for anti-Shank2 and anti-Shank3 antibodies using the whole-cell staining protocol (see previous section).

### Chemical Labeling of Endogenous GluA2-Containing AMPAR Using CAM2

#### Labeling Conditions and Specificity Testing of CAM2 Reagent

For chemical labeling of endogenous GluA2-containing AMPAR in hippocampal neurons, labeling conditions were modified from the original published protocol (Wakayama et al., 2017) to allow for maximal labeling efficiency and minimal internalization of receptors. In brief, neurons were washed twice with Tyrode’s solution [in mM: 96 NaCl, 5.4 KCl, 1 MgCl_2_, 1.8 CaCl_2_*2H_2_O, 10 HEPES and 25 D-glucose, pH 7.3], then incubated in a humidified box for 3 h with 3 μM Alexa 647-CAM2 or Alexa 488-CAM2 in Tyrode’s solution at 17°C to minimize internalization of AMPARs (Wakayama et al., 2017). To assess the specificity of the CAM2 reagent for tagging the GluA2 subunit, after labeling, neurons were washed three times with Tyrode’s solution and then immunostained for GluA1 and GluA2 subunits using the whole-cell staining protocol described previously.

#### Sequential Dual-Labeling Experiment of Endogenous GluA2-Containing AMPAR

Neurons were first labeled with Alexa 647-CAM2 using the labeling protocol described above. Cells were then washed three times with Tyrode’s solution, treated with 10 μM ZnCl_2_ or MgCl_2_ (control) in Tyrode’s solution for 20 min at RT. After treatment, neurons were labeled again with Alexa 488-CAM2. After the second labeling, neurons were washed three times with Tyrode’s solution and immunostained with MAP2 and either Shank2 or Shank3 antibodies using the whole-cell staining protocol described previously.

### Zinc Imaging

#### Labeling

A high affinity zinc-sensitive fluorescent dye, FluoZin-3 AM (*K_d_* for Zn^2+^ ~15 nM), was used for measuring total zinc and a low affinity zinc indicator, Newport Green DCF (NPG, *K*_d_ for Zn^2+^ ~1 μM), for free Zn^2+^. For measuring intracellular zinc, neurons were loaded with FluoZin-3 (1–2 μM) or NPG (5 μM) for 30 min at RT, washed three times with Tyrode’s solution and incubated for another 20–30 min at RT to allow for dye deesterification.

#### Experimental Set Up

To assess free zinc changes with extracellular manipulation of zinc, WT neurons were loaded with NPG as described above. Sister cultures were treated with different ZnCl_2_ concentrations (with or without pyrithione, a zinc ionophore) and TPEN concentration (a zinc chelator) at RT. After 10 min of treatment, neurons were washed with Tyrode’s solution, fixed, washed three times with PBS, and mounted onto slides. To monitor live zinc dynamics, WT neurons were utilized for whole-cell zinc assessment, and neurons pre-transfected with mApple were used for synaptic zinc imaging. After loading with FluoZin-3 as described above, neurons were utilized for time-lapse imaging experiments.

### Image Acquisition

#### Imaging

Pyramidal neurons were selected for image acquisition based on their pyramidal or pear-shaped somata with 3–4 primary dendrites. Three-dimensional fluorescence images (16-bit, 512 × 512) were acquired using MetaMorph 7.0 (Universal Imaging) in conjunction with a Yokogawa CSU 10 spinning disk confocal (Perkin Elmer) fitted on a Zeiss Axiovert 200M inverted microscope. The excitation light of a Krypton/Argon ion laser (643-RYB-A02; Melles Griot) was selected by 488/10 nm, 568/10 nm or 647/10 nm filters (Sutter Lambda filter changer), reflected and then focused through a 63× 1.4 numerical aperture (NA) oil immersion Plan-Apochromat objective lens (Carl Zeiss MicroImaging, Inc.) or a 10× 0.45 NA Plan-Apochromat objective lens. Detection of the fluorescence emission, after passing a 525/50 nm bandpass filter for Alexa 488, a 607/45 nm bandpass filter for Alexa 568 or a 700/75 nm filter for Alexa 647, was obtained using a Cascade 512B camera (Roper). For between sample comparison, all images were acquired with the same settings without knowledge of the experimental condition during image acquisition. To acquire image stacks that could be deconvolved for further analysis, images were sampled using the Nyquist criterion.

#### Time-Lapse Imaging of Zinc Dynamics in Spines

For this experiment, three-dimensional fluorescence images (8-bit, 512 × 512) were acquired using a SP8 laser scanning confocal microscope (Leica Microsystems Inc.) with a white-light laser, hybrid (HyD) photodetectors and a tunable acousto-optical beam splitter. Custom band-passes (FluoZin-3, wavelength: 488 nm, bandwidth: 493–582 nm; mApple, wavelength: 568 nm, bandwidth: 583–602 nm) were set for FluoZin-3 and mApple based on their excitation and emission spectra (FluoZin-3 maxima, Excitation: 494 nm, Emission: 516 nm; mApple maxima, Excitation: 568 nm, Emission: 592 nm). Images were captured with a 63× 1.4 numerical aperture (NA) oil immersion HC PL Apo objective lens (Leica Microsystems Inc.) with a pinhole set at 1 Airy unit. For monitoring spine Zn^2+^ dynamics, images of both mApple and FluoZin-3 channels were captured every 40–60 s. After 5 min of baseline imaging, cells were perfused with high potassium Tyrode’s solution (in mM: 11.5 NaCl, 90 KCl, 1 MgCl_2_, 1.8 CaCl_2_2H_2_O, 10 HEPES and 25 glucose, pH 7.3) for 120–180 s and then washed out with regular Tyrode’s solution for 5–10 min. Zinc increases were measured immediately post-HiK treatment to allow for full exchange of solution.

### Image Analysis

#### Image Preprocessing

Image preprocessing was performed in ImageJ (NIH) unless otherwise specified.

#### 3D Colocalization Analysis for Immunocytochemistry

Images were first preprocessed for analysis. Raw image stacks were background subtracted using a rolling ball radius of 50. They were then 3D deconvolved in Huygens Professional (Scientific Volume Imaging) software using the theoretically calculated point spread functions (PSF) and classic maximum likelihood estimation (CMLE) deconvolution algorithm. Next, primary dendrites were linearized and extracted from the full-frame image using the Straighten plugin for ImageJ.

The identification of protein puncta in straightened dendrites, and analysis of their colocalization in 3D was performed using a custom software package IMFLAN3D—a combination of ImageJ and MATLAB functions (Tai et al., 2007). In brief, straightened dendrites were sharpened, and regions of concentrated fluorophore intensity (puncta) within the images were segmented using a watershed algorithm (ImageJ). Once puncta separation was achieved in each channel, thresholding was done to remove low intensity noise while keeping higher intensities intact. Separate threshold values were determined for each channel, and this common set of threshold values was used to process all images from all conditions in a given experiment.

The resulting images with separated puncta were then processed using custom scripts in Matlab to calculate the properties of the individual 3D puncta. In brief, individual puncta were identified in the binary versions of the raw images using a standard image-processing technique for labeling groups of connected pixels (26-connectivity in 3D). Each separate puncta ended up with a unique label, and this identity information was then used to calculate distributions of individual puncta volume, intensity and other parameters in the raw images. For analyzing the percentage of colocalization between two proteins, the following equation was utilized:

$$\begin{array}{c} \%\text{ protein A overlap}\\ \text{with protein B} \end{array}\}=\frac{\text{Total \# overlapping protein A \& B puncta}}{\text{Total \# protein A puncta}}$$

#### 2D Intensity Analysis of Shank Proteins in Dendritic Puncta vs. Spines

Raw image stacks were processed in Huygens Professional and ImageJ as described above. Deconvolved 3D images of linearized dendrites were then Z-projected (sum intensity). An intensity profile of puncta in the dendritic regions and in the spines were plotted in ImageJ, and the total intensity was quantified using area under the curve integration in Prism 6.0. The ratio of total Shank intensity in the dendritic shaft puncta vs. in the spines were quantified for each Shank protein in each dendrite as a metric for their relative contribution to the immature synapses vs. mature synapses (Niesmann et al., 2011; Valnegri et al., 2011).

#### 2D Density and Fluorescence Intensity Analysis

For quantification of shRNA knockdown efficiency and GluA1/GluA2 expression over development, raw image stacks were background subtracted then Z-projected (average intensity) prior to use of SynPAnal analysis software (Danielson and Lee, 2014) to quantify 2D density and intensity values along primary dendrites. For identification of primary dendrites, the eGFP signal or the GluA1/2 signal was used, and puncta detection was accomplished by thresholding images and counting distinct cluster of four or more adjacent pixels above the intensity threshold. The same detection criteria were applied for different genotypes. Intensity and density values were extracted from the software.

#### 2D Puncta Analysis of CAM2 Labeling of GluA2-Containing AMPAR

Raw image stacks were Z-projected (average intensity). Puncta-by-puncta analysis was performed using OpenView analysis software (Friedman et al., 2000; Arons et al., 2012). Shank2 or Shank3 immuno-positive fluorescent puncta were individually boxed using a Mexican hat filter and then selected based on the following criteria: selected puncta must be above background intensity values in immunostained and CAM2 channels, the puncta must be discrete and non-overlapping with good spatial separation, and the puncta must lie within four pixels of a MAP2-positive dendrite. Puncta fluorescence intensity values were determined, and subsequent data analysis revealed trends in the data.

#### Fluorescence Intensity Analysis of NPG DCF Signal

Raw image stacks were background subtracted and Z-projected (average intensity). Neuronal cells were identified based on morphology using bright field images of the same regions. NeuN staining was performed in an independent set of coverslips to further confirm the neuronal identity of these cells. Puncta intensity values of neuronal somas were then determined using the Time Series Analyzer plugin in ImageJ.

#### Fluorescence Intensity Analysis of FluoZin-3 Dynamics in Spines

Raw image stacks were background subtracted and Z-projected (sum intensity). Primary dendrites were then linearized and extracted from the full-frame image before being analyzed using custom scripts in Matlab (SpineZap; Mysore et al., 2007). To select spines, Z-projected images of FluoZin-3 signal at each time point and of mApple at the first time point were combined into a single stack and Z-projected (sum intensity; referred to as t-projection below). Individual boxes covering the spine and minimal extracellular space were then drawn around each spine so that the morphology of the spine was clearly visible but not in close proximity to axons, dendritic projections or tissue debris. The coordinates of each spine’s box were then utilized to extract time-lapse images from the raw images in order to further verify visually whether or not the selected protrusions were spines. Mean fluorescence intensity values of spines were quantified using custom codes in MATLAB.

#### Statistical Analysis

All statistical tests of imaging data were performed in Prism 6.0. For cumulative probability distributions, each cohort population was composed of a random selection of an equal number of puncta values (50–100) from each cell per condition. The K.S. test was applied to determine statistical significance (*p* < 0.005) for experiments with two samples. Kruskal-Wallis one-way ANOVA with Dunn’s correction *post hoc* multiple comparisons was used in experiments with more than two samples. Two-way ANOVA was used to analyze the developmental difference in the ratio of Shank2 and Shank3 intensities in spines (mature) vs. in dendritic puncta (immature). The Mann-Whitney test was used for comparison of mean values between two non-paired conditions. Wilcoxon matched-pairs signed rank tests were applied to compare between baseline and high potassium conditions in time-lapse experiments and to compare the difference in the ratio of Shank2 and Shank3 intensity in dendritic puncta vs. spine at each time point.

## Results

### Changes in Synaptic Expression of Zinc-Sensitive Shanks and VGluT1 During Development

**Figure 1:**
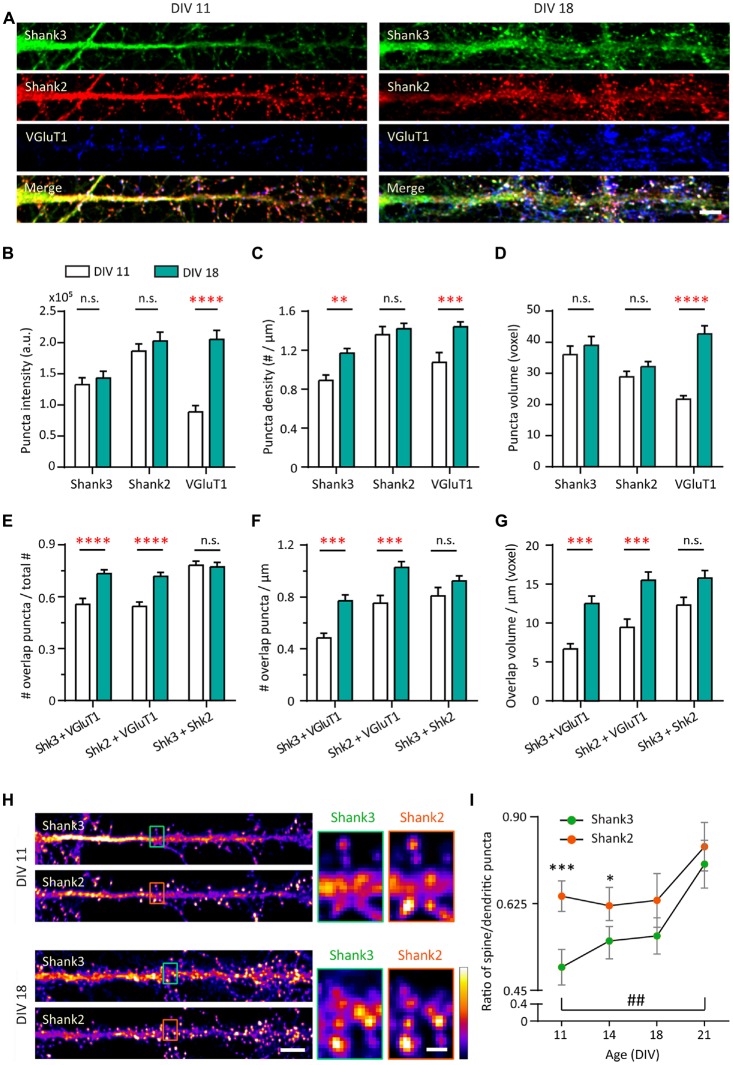
Increase in synaptic expression of Shank2, Shank3 and VGluT1 during development. **(A)** Straightened dendrites of young (days *in vitro* 11, DIV 11) and mature (DIV 18) hippocampal neurons co-immunostained for Shank3 (A488, green), Shank2 (A568, red) and VGluT1 (A647, blue). White puncta in the merge images (bottom) indicate colocalization of all three proteins. Scale bar: 5 μm.** (B–D)** Three-dimensional analysis (IMFLAN3D) of Shank3 and Shank2 puncta at DIV 11 and 18 quantifying intensity **(B)**, density **(C)** or volume **(D)** (mean ± SEM). **(E–G)** Three-dimensional colocalization analysis of pairwise puncta overlap as measured by the ratio **(E)**, density **(F)** and volume **(G)** at DIV 11 and 18 [mean ± SEM; *N* = 16 dendrites from 10 to 12 neurons from two culture preparations (referred to as culture preps from now on; Mann-Whitney tests. n.s. *p* ≥ 0.05, **p < 0.005, ***p < 0.0005, ****p < 0.0001)]. **(H)** Straightened dendrites of young and mature hippocampal neurons immunostained for Shank3 and Shank2. Scale bar: Image, 5 μm; Corresponding inset, 1 μm. **(I)** Summary graphs showing quantification of Shank3 and Shank2 relative enrichment in spines vs. in dendritic puncta (DIV 21, *N* = 18; all others, *N* = 16 dendrites from 10 to 12 neurons from two cultures preps; Kruskal-Wallis one-way analysis of variance (ANOVA) followed by Dunn’s correction *post hoc* multiple comparisons. Comparing between Shank2 and Shank3 at the same age: *p < 0.05; ***p < 0.0005. Comparing Shank3 between different ages: ^##^*p* < 0.005).

To understand the roles of Shank2 and Shank3 on excitatory synaptic development, we compared the developmental changes of endogenous proteins in young (DIV 11) and mature (DIV 18) hippocampal neurons in culture (Figure 1A and Supplementary Figure S1A). Shank postsynaptic clusters (puncta) were analyzed using a three-dimensional blind analysis. Shank2 showed distinct clusters along the dendrites and was present in spines at DIV 11 (Figure 1A, left middle panel). Shank2 puncta remained stable with regard to intensity, density or volume between DIV 11 and DIV 18 (Figure 1A, middle panels and Figures 1B–D). In contrast, at DIV 11 Shank3 was present mainly in the dendritic shaft, especially in the proximal dendrites, and dendritic puncta with limited localization in spines (Figure 1A, left top panel). At DIV 18, the density of Shank3 puncta increased by 31.6% compared to DIV 11 (*p* = 0.0092; Figure 1C) without a change in puncta intensity or volume (Figures 1B,D). Additionally, Shank2 and Shank3 showed strong colocalization (Figure 1E) at DIV 11 (78.14 ± 2.43%) that remained stable through these developmental stages (DIV 18, 77.23 ± 2.58%), implying that both proteins might function in concert at the same synapses. Shank1 was weakly expressed in these cultures during this window (data not shown; Grabrucker et al., 2011) and was excluded from the rest of the study.

Motivated by the localization difference in Shank2 and Shank3 expression pattern at DIV 11 (Figure 1A), we further examined the contribution of each protein to mature vs. immature synapses at two additional time points (DIV 14 and 21). This contribution was measured by the ratio of Shank intensity in spines (mature) vs. in dendritic puncta (immature; Figure 1H; Valnegri et al., 2011). A ratio of zero means that Shank2 and Shank3 exclusively occupy immature synapses on the shaft whereas a ratio of one indicates they contribute equally to both immature synapses on the dendritic shaft and mature synapses on spines. In young neurons (DIV 11 and 14), Shank2 was more biased towards mature synapse localization in spines (0.69 and 0.67) compared to Shank3, which showed a stronger dendritic localization (0.51 and 0.58; *p* = 0.0003 and *p* = 0.0024; Figure 1I). Later in development (DIV 18 and 21), Shank3 localization mimicked Shank2 with an increased spine localization [0.70 at DIV 21 (*p* = 0.0024); Figures 1H,I]. Overall, these findings imply that Shank2 may serve as the primary scaffolding molecule occupying excitatory synapses early in development while Shank3 arrives later, which is consistent with previous studies (Boeckers et al., 1999; Bresler et al., 2004; Grabrucker et al., 2011).

Next, we wanted to understand the development of Shank-containing synapses by also looking at VGluT1 for visualization of presynaptic specializations to distinguish synaptic from non-synaptic puncta. Striking increases in VGluT1 during synapse maturation were seen in all measures (Intensity: 131.8% increase, *p* < 0.0001; Density: 34.078% increase, *p* = 0.0004; Volume: 96.91% increase, *p* < 0.0001), which is consistent with an earlier study (Wilson et al., 2005). Because of these large changes, we again looked at multiple time points (DIV 11, 14, 18 and 21). We found that VGluT1 showed developmental step-like changes in puncta intensity, density and volume between DIV 11 and 21, delineating a clear developmental profile of young neurons (DIV 11–14) and mature neurons (DIV 18–21; Supplementary Figures S1B–E). More than half of Shank2 (54.41%) and Shank3 (55.55%) clusters overlap with punctate VGluT1 staining at DIV 11 (Figure 1E). Concomitant with these developmental VGluT1 changes, significant increases with time were seen in all parameters (fraction, density and volume) of Shank-dependent synapses, as defined by Shank-VGluT1 overlapping puncta (Figures 1E–G).

### Developmental Characterization of AMPAR Structure and Function

**Figure 2:**
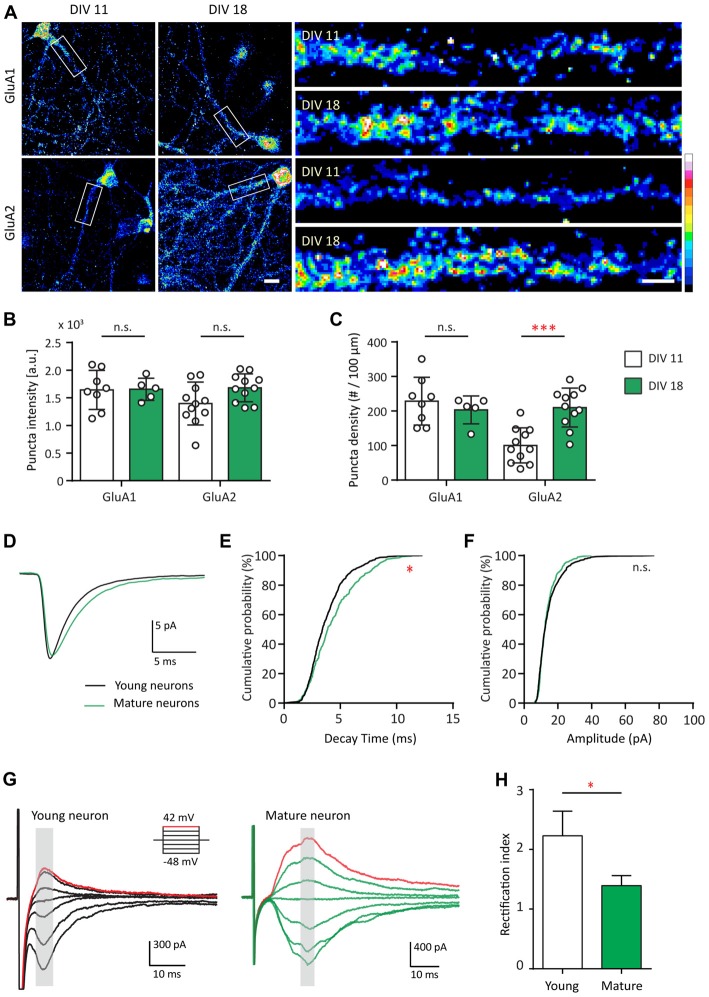
AMPA receptor (AMPAR) subunit composition and functional change during development. **(A)** Hippocampal neurons were fixed and immunostained for GluA1 (top) or GluA2 (bottom) at DIV 11 and DIV 18. White boxes indicate the straightened dendrites (right). Scale bar: image, 15 μm; dendrite, 5 μm.** (B,C)** Summary graphs showing quantification of GluA1 and GluA2 puncta intensity (arbitrary fluorescent units—a.u.) **(B)** and density (puncta per 100 μm; **C**) [mean ± SEM; DIV 11, *N* = 8; DIV 18, *N* = 5 dendrites for GluA1; *N* = 11 for GluA2 from 5 to 10 neurons from two culture preps; Mann-Whitney test. n.s. *p* ≥ 0.05, ***p < 0.001]. **(D)** Ensemble-averaged miniature EPSCs (mEPSCs) from all events recorded in young (black) or mature (green) neurons [*N* = 11 (young) and 10 (mature) neurons from 4 to 7 culture preps, which applies to all subsequent panels unless otherwise specified]. **(E,F)** Cumulative probability histograms of decay time **(E)** or amplitude **(F)** from all isolated events of young or mature neurons [Kolmogorov–Smirnov (K.S.) test, *N* = 600 events (young) and 400 events (for mature), n.s. *p* ≥ 0.005, *p < 0.005]. **(G)** AMPAR-mediated EPSCs (averaged from three trials) evoked at various holding potentials (see diagram, upper right) from a young (black, left) or mature (green, right) hippocampal neuron. Red traces correspond to the current response at the most positive holding potential. Gray shaded bars used for calculating peak current amplitude. **(H)** Summary graph showing the difference of AMPAR inward rectification property between young and mature neurons [mean ± SEM; *N* = 29 cells (young) and 13 cells (mature) from 3 to 5 culture preps. Mann-Whitney test, *p < 0.05].

Since Shank2 and Shank3 serve as master scaffolding molecules that interact with glutamate receptors (Sheng and Kim, 2000; Uchino et al., 2006), we examined whether the developmental changes in AMPAR expression track with those of Shank. Here, we first compared the expression pattern of GluA1 and GluA2 at two different developmental stages (young—DIV 11 and mature—DIV 18) for GluA1 and GluA2 (Figure 2A). GluA1 puncta density and intensity remained the same between DIV 11 and DIV 18 (Figures 2B,C). However, GluA2 puncta density at DIV 18 more than doubled in comparison to that of DIV 11 [100.4 ± 15.31 puncta/μm at DIV 11 vs. 210.1 ± 17.03 puncta/μm at DIV 18 (*p* = 0.004; Figure 2C)]. These data identified the time frame for the AMPAR developmental subunit switch from GluA2-lacking to GluA2-containing receptors in our hippocampal cultures, similar to previous findings (Pickard et al., 2000; Kumar et al., 2002).

Considering the developmental increase of GluA2, we asked whether this contributes to different functional properties of these excitatory synapses. To address this question, AMPAR-mediated miniature EPSCs (mEPSCs) were recorded and compared between both groups of neurons (Figure 2D). AMPAR mEPSC decay time of mature neurons was longer than that of young neurons (*p* ≤ 0.005; Figures 2D,E), a finding consistent with earlier work (Brill and Huguenard, 2008). No difference was found between young and mature neurons in terms of AMPAR mEPSC amplitude (Figures 2D,F) or frequency (data not shown). The developmental increase in decay time could be explained by the slower decay kinetics of GluA2-containing AMPAR (Geiger et al., 1995).

GluA2-lacking AMPARs are more sensitive to polyamine blockage at positive holding potentials and thus pass less outward current than inward current at equivalent distance from the reversal potential (Kumar et al., 2002). Therefore, we investigated whether inward rectification of electrically evoked AMPAR EPSCs changed during development. This was accomplished by measuring the AMPAR evoked response at different holding voltages (Figure 2G). In young neurons, AMPAR EPSCs were consistently smaller at positive holding potentials compared with those at corresponding negative potentials (Figure 2G, left). In mature neurons, AMPAR EPSCs were similar in magnitude at equipotential levels on either side of AMPAR reversal potential (~3 mV in our experimental condition; Figure 2G, right), consistent with decreased rectification. For comparison, we employed a rectification index (RI) defined as the ratio of AMPA conductance measured at corresponding positive over negative holding potentials. The RI of young neurons was larger than that of the mature neurons (Figure 2H, 37.66%, *p* = 0.04). Thus, in young neurons, AMPAR-EPSCs were mostly characterized by inward rectification in contrast to mature neurons, similar to previous observations (Jonas et al., 1994; Geiger et al., 1995; Kumar et al., 2002; Brill and Huguenard, 2008; Bariselli et al., 2016). Together, these data indicate that AMPAR in young and mature neurons are fundamentally different in terms of their subunit compositions, leading to functional effects on their decay kinetics and inward rectification property.

### Depolarization and Exogenous Zinc Cause an Increase in Intracellular Zinc

Due to the similar developmental expression profiles of GluA2 and synaptic Shank2 and Shank3 (Figures 1, 2), we hypothesized that these Shank proteins could play a role in regulating AMPAR subunit composition during development. Previous work from our lab showed that Shank3 is required for zinc-induced AMPAR synaptic potentiation (Arons et al., 2016), Thus, zinc influx into the postsynaptic compartment could activate Shank-dependent regulation of AMPAR subunit composition. We next examined if neuronal stimulation affects the dynamics of postsynaptic zinc, which could enter from presynaptic-released zinc in the cleft or postsynaptic sources (Masters et al., 1994; Cole et al., 2000; Lee et al., 2003; Bossy-Wetzel et al., 2004; Frederickson et al., 2006).

**Figure 3:**
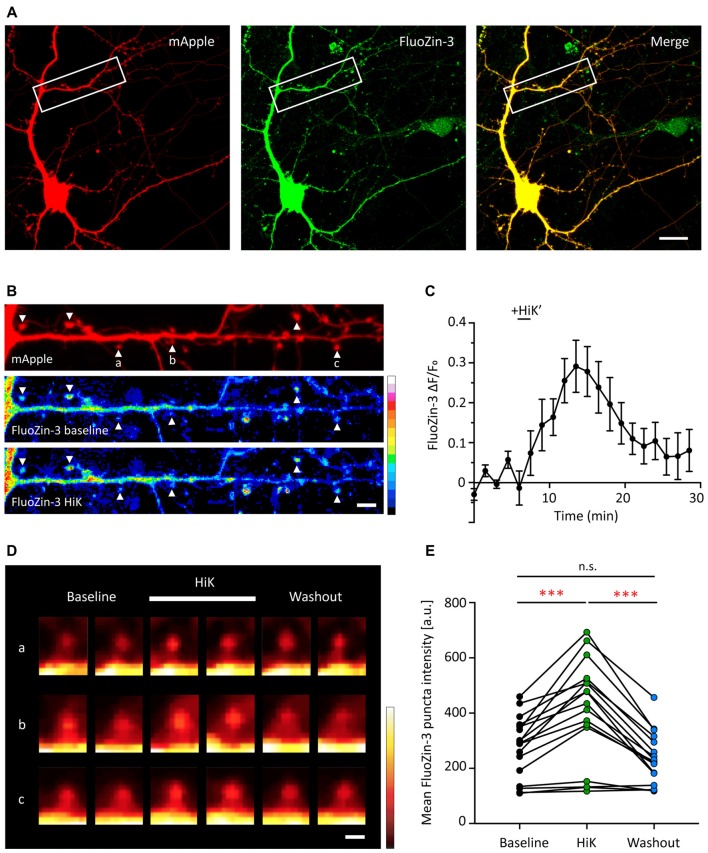
Depolarization induces a transient and reversible increase of zinc in postsynaptic spines. **(A)** A young hippocampal neuron (DIV 14) transfected with mApple (left, red) and loaded with FluoZin-3 (middle, green). mApple was used as a morphological marker to indicate dendrites and postsynaptic spines. Yellow indicates clear colocalization in the merge image (far right). White boxes mark the dendrite shown in **(B)**. Scale bar: 10 μm. **(B)** Straightened dendrite with mApple (top) and FluoZin-3 at baseline (middle) and during depolarization conditions [bottom; 90 mM KCl (HiK), 120–180 s]. White arrowheads mark spines being quantified in **(C)** and shown in (**D**; indicated by a–c). Scale bar: 5 μm. **(C)** Time course of changes in FluoZin-3 (*ΔF/F_o_*) with depolarization for spines in **(B)**. Black line indicates when depolarization was applied (HiK’). Mean ± SEM for *N* = 6 spines in one dendrite, shown in **(B)**. **(D)** FluoZin-3 fluorescence changes in individual spines (a–c) under baseline, HiK (white line) and washout conditions indicated in **(B)**. Scale bar: 1 μm. **(E)** Effects of depolarization on FluoZin-3 in spines of individual hippocampal neurons averaged across baseline, during HiK and washout conditions (one-way ANOVA followed by Sidak’s multiple comparisons, *N* = 17 dendrites from five neurons from four culture preps, n.s. *p* > 0.05, ***p < 0.001).

Dissociated hippocampal neurons were transfected with mApple, as a structural marker, and then loaded with the membrane permeable fluorescent zinc indicator, FluoZin-3-AM (Figure 3A). FluoZin-3 fluorescence was present throughout the soma, dendrite and spines and co-localized with mApple (Figures 3A,B). Importantly, FluoZin-3 allows for the detection of small changes in intracellular zinc (*K_d_* = 1.5 nM, detection range 10 nM-300 μM) and is unaffected by millimolar concentration of calcium (Zhao et al., 2008). In the absence of exogenous stimulation, the FluoZin-3 fluorescence signal remained stable over time (Figure 3C). To determine whether neural activity affects postsynaptic zinc levels, we briefly depolarized neurons by the application of 90 mM high potassium stimulation (HiK). Here, we observed a transient increase of FluoZin-3 intensity in spines (Figures 3C–E, 47.75% increase between baseline and during HiK, *p* = 0.0001). The elevation was fully reversible after washout (Baseline vs. Wash, *p* > 0.05; HiK vs. Wash, *p* = 0.0001). Together, these data show that zinc is elevated in postsynaptic spines during neuronal depolarization, which could bind to Shank2 and Shank3 and influence the activation of these proteins via conformational changes (Arons et al., 2016).

We then examined how extracellular manipulations of zinc change intracellular zinc levels (DIV 11–30). Here, Newport Green DCF (NPG) was used due to its lower affinity to zinc (*K_d_* = 1.5 μM, detection range 1 μM-1 mM) for detection of free intracellular zinc (Thompson et al., 2002). With the application of 300 μM ZnCl_2_, hot spots of NPG appeared along dendrites after a 10 min incubation, potentially indicating free zinc accumulation in synaptic puncta (Supplementary Figure S2A). Treatment with ZnCl_2_ in the presence of a zinc ionophore, pyrithione (MNO), allowed the passive transport of zinc between the intra- and extracellular milieu and further induced the appearance of these putative zinc synaptic puncta (Supplementary Figure S2A). To assess the free zinc at baseline condition, we used a high affinity zinc chelator, TPEN (50 μM; *K*_d_ = 0.7 fM; Radford and Lippard, 2013). The zinc-chelating effect of TPEN was confirmed by its capacity to reduce somatic FluoZin-3 signal (data not shown). In contrast, we observed no quantifiable difference of NPG signal between baseline and zinc chelator (TPEN, 50 μM) treatment. This result showed that minimal, if any, free intracellular zinc is detectable by this method under baseline conditions (Supplementary Figure S2C), consistent with previous studies (Sensi et al., 1997; Canzoniero et al., 1999). At the population level, 10 μM extracellular ZnCl_2_ application increased somatic NPG signal above baseline by 24.91% (Supplementary Figures S2B,C), and further increasing ZnCl_2_ concentration to 300 μM raised the intracellular NPG signal even higher. Taken together, these results highlight the low levels of free intracellular zinc and that exogenous addition of ZnCl_2_ can elevated this concentration above baseline. Based on these findings, we established our experimental conditions for further experiments using 10 μM ZnCl_2_.

### Zinc Treatment Enhances AMPAR-Mediated Synaptic Responses in Young Neurons

**Figure 4:**
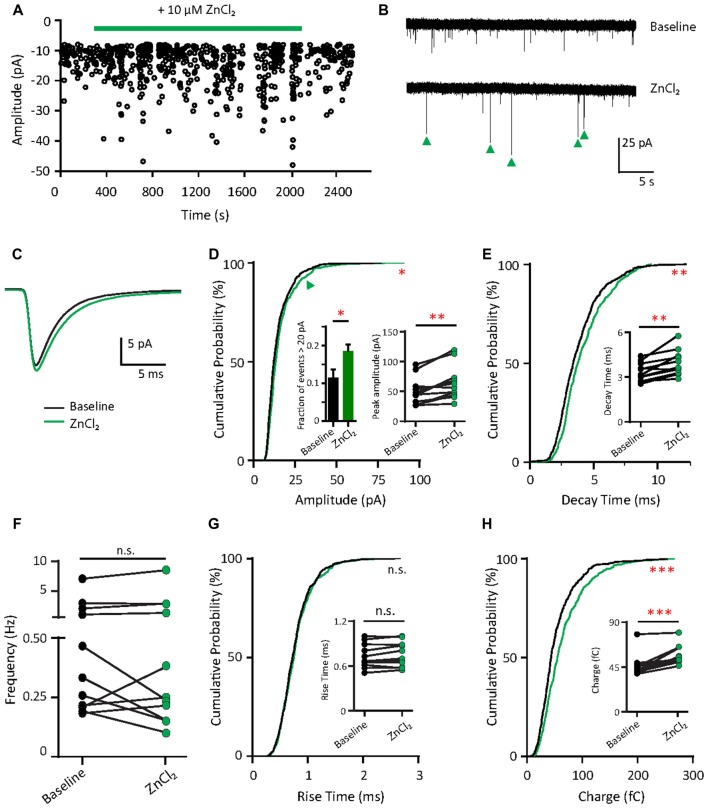
Zinc treatment enhances synaptic efficacy of AMPAR mEPSCs in young neurons. **(A)** AMPAR mEPSCs were recorded from a young hippocampal neuron (DIV 11). Green bar: 10 μM ZnCl_2_ application. **(B)** AMPAR mEPSC recording traces from the neuron shown in **(A)** at baseline (top) and during ZnCl_2_ (bottom). Green arrowheads mark the events with larger amplitude during ZnCl_2_ treatment. **(C)** Ensemble-averaged mEPSCs from the baseline and zinc conditions measured in the same neurons (baseline: black, ZnCl_2_: green, *N* = 11 cells from seven culture preps, which applies to all subsequent panels unless otherwise specified). **(D,E,G,H)** Cumulative probability histograms of amplitude **(D)**, decay time **(E)**, rise time **(G)** and charge **(H)** of isolated events from baseline (black) and ZnCl_2_ (green) conditions (K.S. test, *N* = 600 events. n.s. *p* ≥ 0.005, *p < 0.005, **p < 0.001, ***p < 0.0001). First inset in **(D)**: summary graph showing the difference of fraction of events with amplitude >20 pA between baseline and ZnCl_2_ conditions. All other insets show per-cell-basis pairwise comparison of amplitude **(D)**, decay time **(E)**, rise time **(G)** and charge **(H)** between baseline and ZnCl_2_ conditions (Wilcoxon test, n.s. *p* ≥ 0.05, **p < 0.01, ***p < 0.001). **(F)** Pairwise comparison of AMPAR mEPSC frequency between baseline and ZnCl_2_ conditions (Wilcoxon test, n.s. *p* ≥ 0.05).

What are the functional consequences of the elevation of synaptic zinc? To test the functional effects of zinc on AMPAR, we recorded mEPSCs from young hippocampal neurons (DIV 11–14; Figure 4A). After a 10 min application, the addition of 10 μM ZnCl_2_ was associated with a relative increase (39.69%, *p* = 0.0186) in a fraction of large amplitude events (event > 20 pA) and a consistent increase of peak amplitude (29.93%, *p* = 0.002; Figures 4B–D). However, the frequency of AMPAR mEPSCs was unaffected by zinc application (Figure 4F), suggesting that the effect of zinc is likely postsynaptic. Consistent with a postsynaptic locus of action, zinc lengthened the decay times of AMPAR mEPSCs (19.76%, *p* = 0.002; Figures 4C,E) but did not affect rise time (Figure 4G). The increased amplitude and lengthened decay time resulted in increased synaptic efficacy as measured by the charge transfer (22.75%, *p* = 0.001; Figure 4H). These results reveal that zinc enhanced the strength of AMPAR-mediated synaptic transmission in young neurons, perhaps via activity at postsynaptic sites affecting AMPAR composition and hence response amplitude and kinetics.

### Zinc Treatment Reduces Inward Rectification Property of AMPARs in Young Neurons

**Figure 5:**
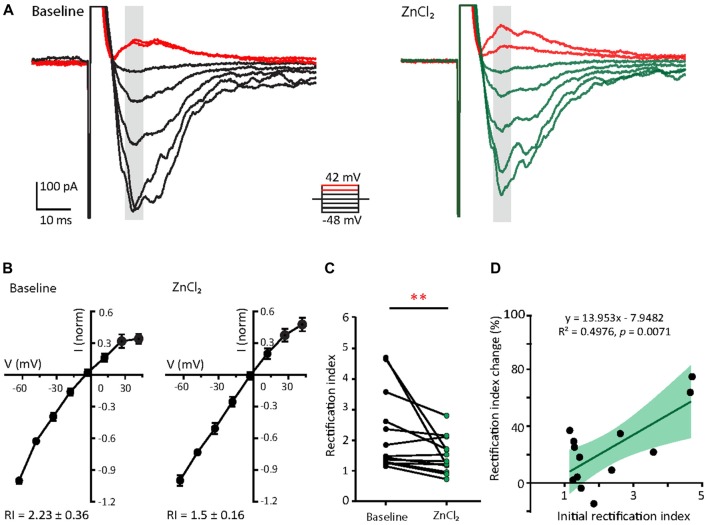
Zinc treatment decreases AMPAR inward rectification in young neurons. **(A)** AMPAR-mediated EPSCs (averaged from three trials) evoked at various holding potentials [diagram (middle)] from a young hippocampal neuron during baseline (left) and after 10 min of 10 μM ZnCl_2_ application (right). Red traces correspond to the current response at the two most positive holding potentials. Gray shaded bars used for calculating peak current amplitude in **(B)**. **(B)** Normalized current-voltage relationship of the pooled data recorded from the same neurons at baseline (left) and ZnCl_2_ treatment (right; mean ± SEM for *N* = 13 cells from five culture preps, which applies to all subsequent panels unless otherwise specified). **(C)** Summary graph showing effect of ZnCl_2_ application on the rectification indices (RI) of young neurons (Wilcoxon test, **p < 0.01). **(D)** Relationship between initial RI (during baseline) and the magnitude of RI change with ZnCl_2_ application (Pearson correlation, **p < 0.01). Green shaded area indicates the 95% confidence interval region.

Since the decay time constant increases as a function of GluA2 content (Figure 2; Geiger et al., 1995), the zinc-induced slowing of decay might be due to the recruitment of GluA2. To assess how zinc alters the subunit composition of functional AMPARs in young neurons, we indirectly assessed GluA2 content by measuring the current to voltage relationship of evoked AMPA EPSCs (evoked EPSCs) at baseline and during zinc treatment. At baseline, synaptic currents were reliably smaller at positive holding potentials in comparison to those at corresponding negative levels, indicating these receptors were inwardly rectifying. The application of zinc led to a more linear current to voltage relationship (Figures 5A,B, right panels) and a reduction in the RI (32.8%, *p* = 0.0061; Figure 5C), observed after 10 min of treatment. Since more linear I/V curves are indicative of higher relative proportions of GluA2-containing AMPAR (Kumar et al., 2002; Brill and Huguenard, 2008), this result suggests that zinc specifically recruited receptors containing GluA2 subunits and/or reduced the synaptic level of GluA2-lacking AMPAR.

We also observed that AMPARs in young neurons have a wide range of RI (1.15–4.71) at baseline, implying variable basal synaptic GluA2 content. Motivated by this observation, we next examined the relationship between initial RI and magnitude of change induced by zinc. From this analysis, the magnitude of RI changes showed a strong positive correlation with the initial RI (Figure 5D; *R*^2^ = 0.498, *p* = 0.0071). These data show that the initial GluA2 content affects the sensitivity of AMPAR to zinc application.

### AMPARs of Mature Neurons Are Not Affected by Zinc Treatment

**Figure 6:**
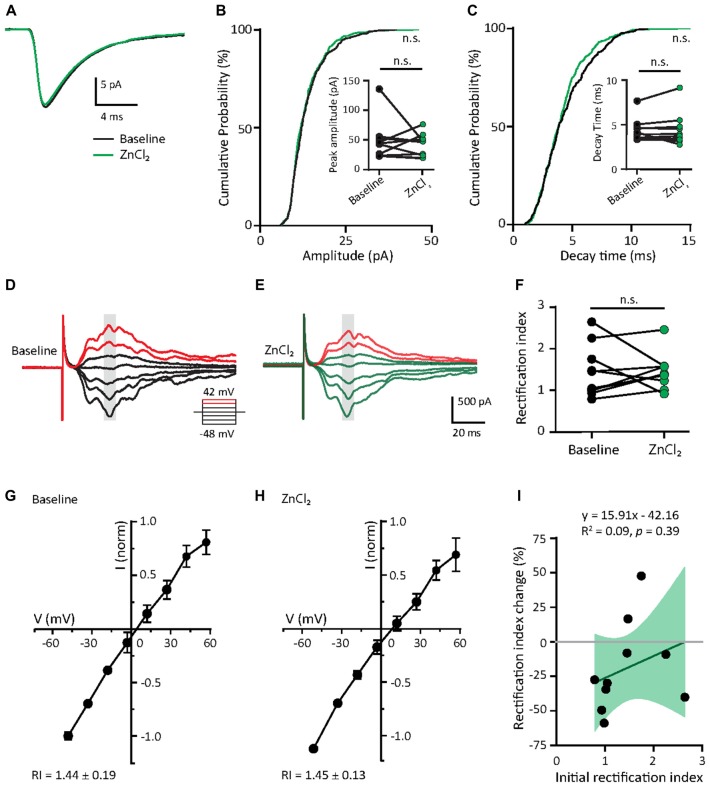
Zinc treatment does not affect miniature or evoked AMPAR EPSCs in mature neurons. **(A)** Ensemble-averaged mEPSCs from the baseline and zinc conditions recorded in mature neurons (baseline: black, ZnCl_2_: green, *N* = 10 cells). **(B,C)** Cumulative probability histograms of amplitude **(B)** or decay time **(C)** of isolated events from baseline and ZnCl_2_ conditions (K.S. test, *N* = 400 events from 10 cells from four culture preps per condition, n.s. *p* ≥ 0.05). Insets display per-cell-basis pairwise comparison of amplitude **(B)** and decay time (**C**; Wilcoxon test, *N* = 10 cells, n.s. *p* ≥ 0.05). **(D,E)** AMPAR-mediated EPSCs (averaged from three trials) evoked at various holding potentials [diagram (middle)] from a mature hippocampal neuron (DIV 18) during baseline **(D)** and after 10 min of 10 μM ZnCl_2_ application **(E)**. Red traces correspond to the current response at the two most positive holding potentials. Gray shaded bars mark the regions used for calculating peak current amplitude in **(G,H)**. **(F)** Summary graph showing effect of ZnCl_2_ application on RI of mature neurons (Wilcoxon test, *N* = 10 cells from three culture preps, which applies to all subsequent panels unless otherwise specified; n.s. *p* ≥ 0.05). **(G,H)** Normalized current-voltage relationship (mean ± SEM) of the pooled data recorded from the same neurons at baseline **(G)** and ZnCl_2_ treatment **(H)**. **(I)** Relationship between the initial RI (during baseline) and the magnitude of RI change with ZnCl_2_ application (Pearson correlation, n.s. *p* ≥ 0.05). Green shaded area indicates the 95% confidence interval.

Given that the initial GluA2 content appears to predict the response to zinc treatment (Figure 5D) and mature neurons had higher GluA2 puncta density than young neurons (Figures 2A–C), we hypothesized that mature neurons would have limited sensitivity to zinc. We then recorded AMPAR mEPSCs in mature neurons (DIV 18–23) at baseline and examined the effect of zinc application. No detectable changes in any AMPAR mEPSC parameters (amplitude, kinetics or frequency) in mature neurons were observed with zinc application (Figures 6A–C and Supplementary Figures S3A–E), indicating that zinc has no significant influence on unitary synaptic strength, kinetics or active synapse numbers at this age of neuronal development. The insensitivity of AMPAR mEPSCs in mature neurons to zinc application is unlikely due to a ceiling effect since no difference in amplitude between young and mature neurons was observed (Figure 2F). Increasing zinc concentrations up to 30 μM also did not elicit any effect on AMPAR mEPSCs (data not shown). These results indicate that the insensitivity of AMPAR mEPSCs in mature neurons is independent of the availability of zinc.

Since zinc did not affect the decay kinetics of AMPAR mEPSCs, we predicted that it also would not alter the subunit composition of AMPARs. To indirectly test this, we assessed the rectification property, which would reflect the relative contribution of GluA2 to synaptic EPSCs (Figures 6D,E,G,H). In mature neurons, AMPAR-evoked EPSCs had a highly linear I/V relationship and low inward rectification (RI = 1.44 ± 0.19), similar to Figures 2G,H. This low IR remained unchanged with zinc application in these cells, suggesting that zinc exerted no effect on AMPAR subunit compositions (Figures 6D–H). As a result, no correlation was seen between initial RI and the magnitude of change induced by zinc treatment. Taken together, the addition of zinc had no significant effect on AMPAR function and subunit composition in mature neurons, likely due to the high basal level of GluA2-content in these cells (Figure 2C).

### Zinc Recruits Surface GluA2-Containing AMPARs and Disperses GluA1 at Existing Shank-Positive Puncta

**Figure 7:**
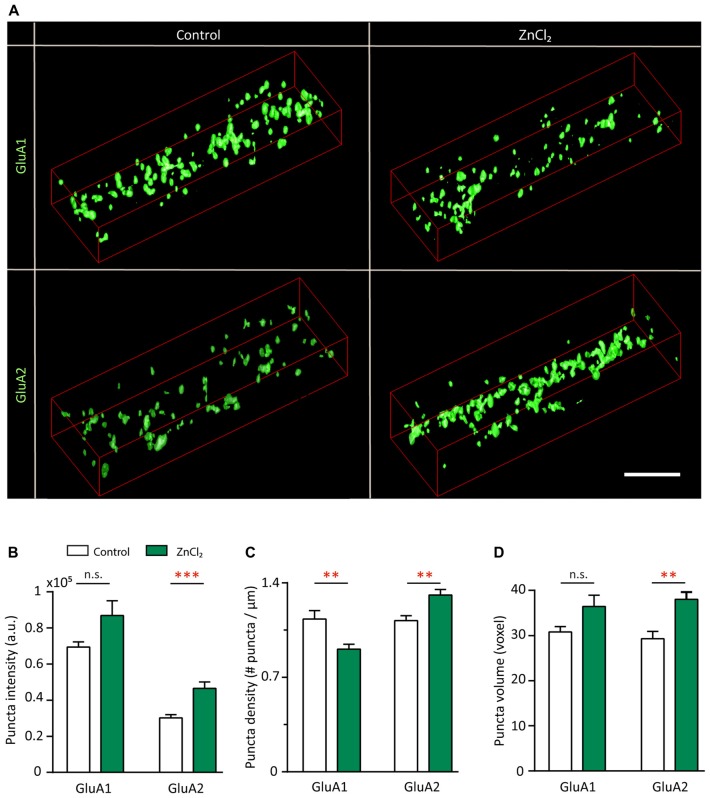
Zinc treatment recruits surface GluA2 and disperses surface GluA1. **(A)** Straightened dendrites from young hippocampal neurons (DIV 14) treated with control (left) or 10 μM ZnCl_2_ (right) conditions. Neurons were live surface labeled for GluA1 (top) or GluA2 (bottom). Scale bar: 4 μm. **(B–D)** Summary graphs showing quantification of GluA1 and GluA2 puncta intensity **(B)**, density **(C)** and volume **(D)** [mean ± SEM; Mann-Whitney test for GluA1, *N* = 19 (control), 22 (ZnCl_2_) dendrites from 10 to 14 neurons from two culture preps; for GluA2, *N* = 24 dendrites from 10 to 14 neurons from two culture preps; n.s. *p* ≥ 0.05, **p < 0.01, ***p < 0.001].

To understand the subunit composition changes induced by zinc, we examined the localization of GluA1 and GluA2 using an antibody directed against an extracellular epitope for each subunit (Figure 7A). Sister cultures were treated with either control or zinc conditions, immunolabeled and analyzed blind in three-dimensions for surface GluA1 or GluA2. This served as an immunocytochemical index of AMPAR subunit composition for comparison with mini and evoked recordings (Lu et al., 2001; Thiagarajan et al., 2005; Kalashnikova et al., 2010). Under control conditions, numerous, bright GluA1 clusters decorated the dendrite (Figure 7A, top left) in comparison to the low intensity levels of GluA2 puncta (Figure 7A, bottom left). Zinc treatment led to a marked increase of GluA2 puncta in terms of intensity (54.34%, *p* = 0.0001), volume (29.8%, *p* = 0.0077) and density (18%, *p* = 0.0016; Figures 7B–D). The modest change in density suggests that zinc has a stronger effect on GluA2 at preexisting synapses. This agrees with our findings in which zinc lengthened AMPAR mEPSC decay time and increased amplitude but did not affect frequency (Figure 4). Zinc also induced a reduction (19.6%, *p* = 0.0097) of GluA1 puncta density (Figure 7C), similar in magnitude to the increase in GluA2 density. This further emphasized that the major effect of zinc was to alter AMPAR subunit composition from GluA2-lacking to GluA2-containing at existing synapses.

**Figure 8:**
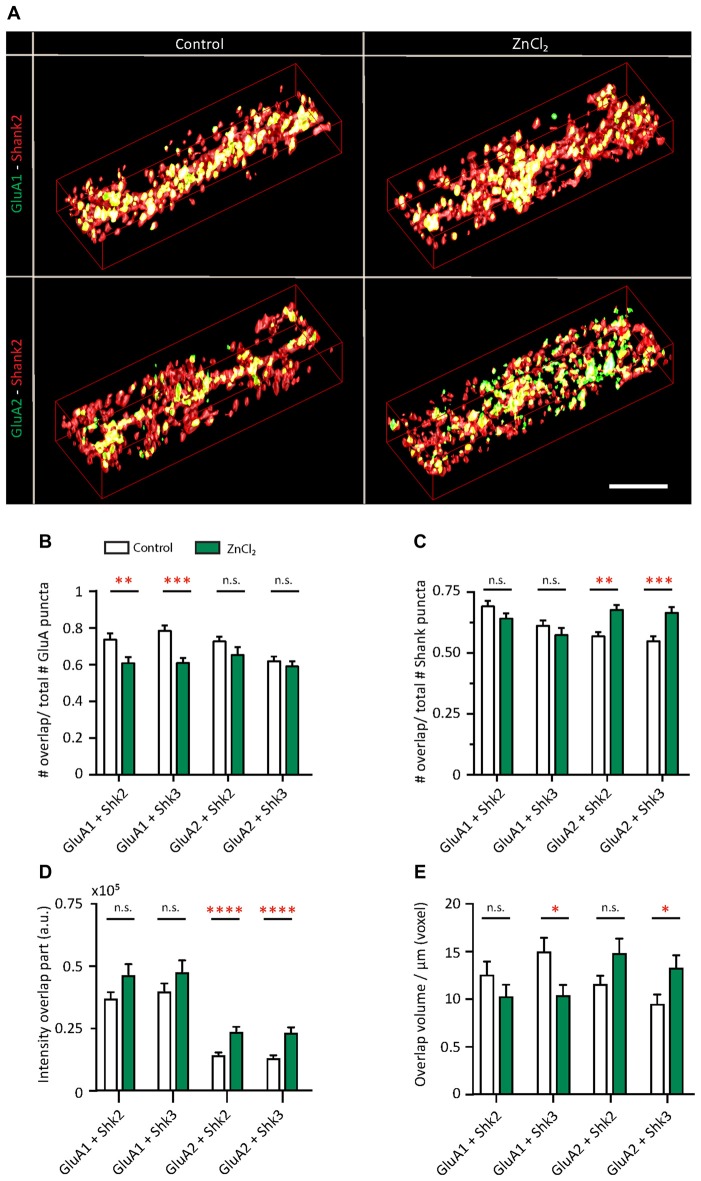
Zinc treatment alters the colocalization between AMPAR subunits and Shank. **(A)** Straightened dendrites from young hippocampal neurons (DIV 14) treated with control (left) or 10 μM ZnCl_2_ (right). Neurons were live surface labeled for GluA1 (green; top) or GluA2 (green; bottom) before being fixed and co-immunostained for both Shank2 (red) and Shank3 (not shown). Yellow indicates colocalization. Scale bar: 4 μm.** (B–E)** Three-dimensional colocalization analysis of pairwise puncta overlap using IMFLAN3D as measured by the fraction of overlap **(B,C)**, intensity **(D)** and volume **(E)** for baseline and ZnCl_2_ conditions (mean ± SEM) [Mann-Whitney test, for GluA1 + Shank3 or Shank2, *N* = 19 (control) and 22 (ZnCl_2_) dendrites from 10 to 14 neurons from two culture preps; for GluA2 + Shank3 or Shank2, *N* = 24 dendrites from 10 to 14 neurons from two culture preps; for Shank2 + Shank3, *N* = 43 (control) and 46 (ZnCl_2_) dendrites from 20 to 25 neurons from four culture preps; n.s. *p* ≥ 0.05, *p < 0.05, **p < 0.01, ***p < 0.001, ****p < 0.0001].

Do the zinc-induced changes of AMPAR subunit composition involve Shank2 and Shank3? We looked at the pattern of surface GluA1 and GluA2 incorporation at (Figure 8B, Supplementary Figure S4) Shank-positive puncta with the same three-dimensional analysis described previously (Figure 1). The distribution of AMPARs and Shank puncta were analyzed (defined as overlap of GluA1 or GluA2 with Shank-positive or non-Shank puncta; Figure 8). A significant fraction of GluA2-positive puncta contained Shank2 (72.69%) and Shank3 (61.89%; Figure 8B, Supplementary Figure S4). A similar fraction of GluA1-positive puncta associated with Shank2 (73.55%) and Shank3 (78.38%) puncta (Figure 8B). This suggests that the majority of AMPAR (both GluA1 and GluA2 clusters) were found at Shank-positive puncta, consistent with previous findings on the interaction between both subunits with Shank2 and Shank3 (Sheng and Kim, 2000; Uchino et al., 2006). On the other hand, just over half of all Shank puncta were GluA2-positive (Shank2 56.76%; Shank3 54.82%; Figure 8C) with a higher fraction associated with GluA1 (Shank2 69.18%; Shank3 61.17%; Figure 8C).

Interestingly, with zinc treatment, there was a significant decrease in the fraction of GluA1 overlapping with Shank2 and Shank3 with a large concurrent increase of GluA1 at non-Shank sites (Figure 8B and Table 1). In contrast with regard to the total GluA2 population, there was no change in the fraction of overlap at any type of Shank or non-Shank puncta with zinc treatment. Instead, with regard to Shank puncta, zinc changed the distribution of GluA2, such that a higher fraction of Shank co-clustered with GluA2 than in control conditions (Table 1). A significant concomitant decrease in GluA2 was seen at both non-Shank2 and non-Shank3 sites (Table 1). Together, these data suggest that zinc preferentially recruits GluA2 to Shank puncta and disperses GluA1 to non-Shank sites.

**Table 1:** Effect of zinc on GluA—Shank colocalization.

		Intensity of overlap	Volume of overlap/μm	Fraction of overlap/total GluA	Fraction of overlap/total Shank
**GluA1**	**Shank2**	n.s.	n.s.	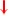 17.4% (0.0074)	n.s.
	**Shank3**	n.s.	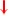 30.51% (0.0209)	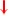 22.34% (0.0002)	n.s.
	**Non-Shank2**	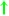 132.65% (<0.0004)	n.s.	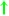 48.39% (0.0074)	n.s.
	**Non-Shank3**	n.s.	n.s.	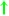 80.99% (0.0002)	n.s.
**GluA2**	**Shank2**	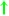 64.05% (<0.0001)	n.s	n.s.	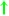 19.22% (0.0010)
	**Shank3**	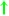 75.04% (<0.0001)	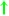 40.19% (0.0153)	n.s.	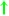 21.32% (0.0008)
	**Non-Shank2**	n.s.	n.s.	n.s.	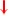 25.23% (0.0010)
	**Non-Shank3**	n.s.	n.s.	n.s.	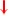 25.87% (0.0008)

*Values are expressed as % change of zinc condition in comparison to control, (p value). For p > 0.05, the values of changes are written as “n.s.”. Red and green arrows indicate the direction of changes (Red arrow: Decrease, Green arrow: Increase)*.

Besides increasing the clustering density of GluA2 and Shank3, zinc treatment also led to an increase in fluorescence intensity (75.04%) and volume (40.19%) for GluA2 specifically at Shank3-positive puncta (Figures 8D,E), further supporting the hypothesis that zinc selectively induces GluA2 incorporation to Shank3-positive puncta. A similar increase in intensity in response to zinc was seen in GluA2 at Shank2 puncta (64.05%) with no significant change in volume (Figures 8D,E and Table 1). In contrast, zinc decreased GluA1 overlap with Shank3 as measured by volume (Figure 8E and Table 1). A significant concomitant increase in intensity of GluA1 (132.65%; *p* < 0.0004) was seen at non-Shank2 sites (Table 1), perhaps corresponding with non-synaptic locations since Shank2 and Shank3 showed near 100% colocalization with other postsynaptic markers (Homer and PSD95) at this age (data not shown).

These findings implicate Shank2 and Shank3 as key players in the AMPAR subunit switch induced by zinc. Our treatment protocol did not elicit any changes of Shank2 or Shank3 puncta intensity, volume or density (data not shown), implying that zinc mainly affects Shank activation (Arons et al., 2016) in association with a change in binding preference from GluA1 to GluA2.

### Zinc-Sensitive Recruitment of GluA2 via Exocytosis and Lateral Diffusion Is Mediated by Shank3

To further understand which cellular processes underlie the zinc-dependent recruitment of GluA2, we performed a sequential dual-labeling experiment to monitor both lateral diffusion and exocytosis with zinc treatment. Here, we used a recently developed method to visualize native GluA2 using chemical AMPAR modification (CAM2) reagents that allow for covalent chemical labeling with a small fluorophore (Alexa fluors; Wakayama et al., 2017). Control experiments were performed to confirm the specificity and saturation binding of all surface GluA2 for this dual-labeling experiment in our neuronal culture system. Similar to the original study (Wakayama et al., 2017), we found that 80%–90% of CAM2 puncta colocalized with GluA2 staining, whereas there was limited colocalization between CAM2 and GluA1 (data not shown). We then next tested varying concentrations of CAM2 for labeling and chose an excess concentration (3 μM) for subsequent experiments to ensure saturated labeling of surface GluA2.

**Figure 9:**
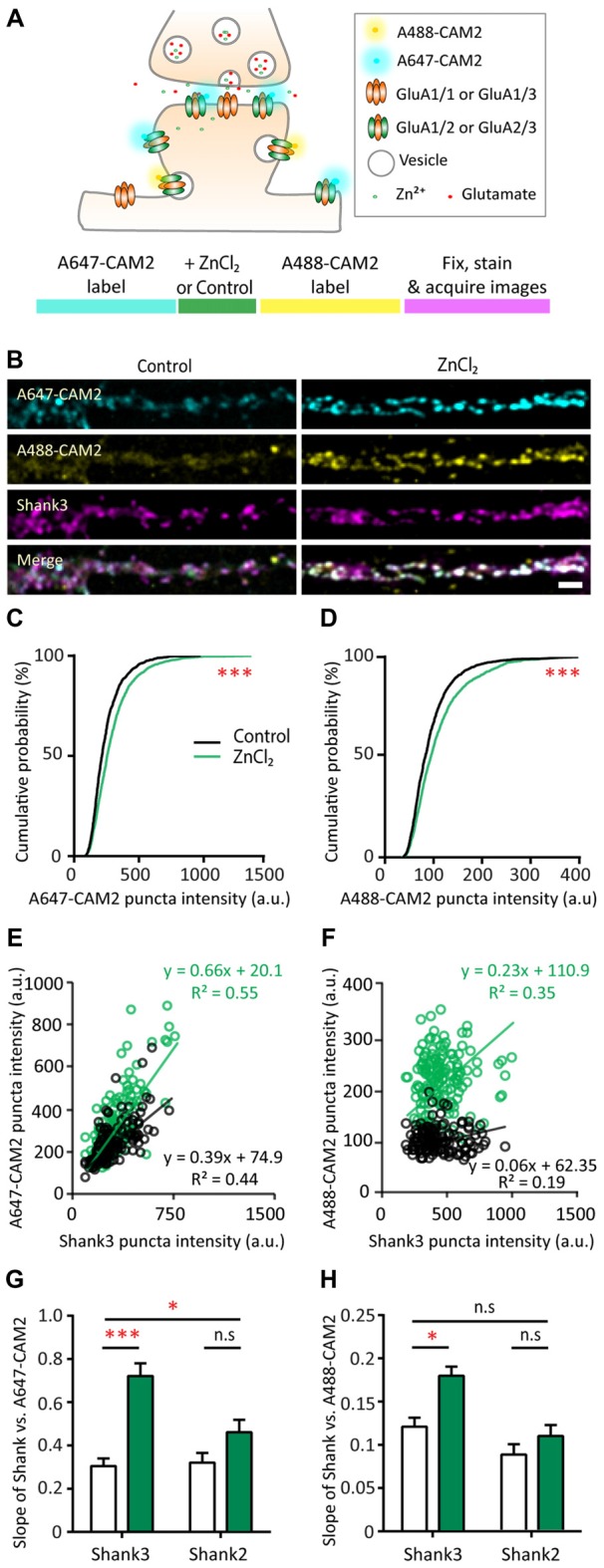
Probing mechanisms of zinc-dependent AMPAR trafficking with CAM2. **(A)** Schematic of sequential dual chemical labeling of GluA2 subunit of AMPAR using A647-CAM2 and A488-CAM2 reagents (top). Sequence of the labeling experiment using CAM2 to probe zinc-dependent AMPAR trafficking (bottom). **(B)** Straightened dendrites of hippocampal neurons were pre-labeled with A647-CAM2 (top), then treated with either control (10 μM MgCl_2_, left) or 10 μM ZnCl_2_ (right) conditions before being labeled with A488-CAM2 (second panel from the top), and finally fixed and stained for Shank3 (third panel from the top) and Shank2 (not shown). White puncta in the merge images (bottom) indicate colocalization of all three signals. Scale bar: 5 μm. **(C,D)** Cumulative probability histograms of puncta intensity from control and ZnCl_2_ conditions for A647-CAM2 **(C)** and A488-CAM2 **(D)** signal (K.S., *N* ~ 1500 puncta from 25 (control) and 30 (ZnCl_2_) cells from three culture preps; ***p < 0.0001). **(E,F)** Example of fluorescence intensities of individual colocalized CAM2 puncta (A647 or A488) plotted as a function of the corresponding colocalized Shank3 puncta intensities for two neurons. Comparisons were made between sister cultures. Linear regression is shown as a solid line for each condition (black = control; green = zinc). **(G)** Summary graph showing comparison of the slopes of linear regressions of puncta intensity between Shank3 or Shank2 and A647-CAM2 from control (white) and ZnCl_2_ (green) conditions. **(H)** Similar to G but for Shank3 or Shank2 and A488-CAM2 [two-way ANOVA, Shank2 co-stained neurons, *N* = 15 (control) and 20 (ZnCl_2_) cells from 3 to 4 coverslips from two culture preps; Shank3 co-stained, *N* = 10 cells from 3 to 4 coverslips from two culture preps; n.s. *p* ≥ 0.05, *p < 0.05. Sidak correction multiple *post hoc* comparisons; n.s. *p* ≥ 0.05, *p < 0.05, ***p < 0.001].

In order to examine AMPAR dynamics with zinc, surface GluA2 subunits were initially labeled with Alexa 647-CAM2 for saturated labeling. Neurons were then treated with control or ZnCl_2_ conditions, which could recruit receptors from outside of the synapse and increase synaptic Alexa 647-CAM2 signal. If exocytosis was enhanced with zinc treatment, these new surface receptors would be labeled during the second labeling with Alexa 488-CAM2. Shank2 or Shank3 were also labeled as postsynaptic markers and used to understand their roles in zinc-sensitive AMPAR dynamics (Figure 9A).

To this end, we measured the fluorescence intensity in both CAM2 channels at Shank-positive puncta. Using puncta-by-puncta analysis (Friedman et al., 2000; Arons et al., 2012), we found that both lateral diffusion and exocytosis were involved with the zinc-dependent trafficking of GluA2 to synaptic sites as measured by the increase of both A647-CAM2 (16.61%, *p* < 0.0001) and A488-CAM2 puncta intensity (14.1%, *p* < 0.0001; Figures 9B–D and Supplementary Figure S5A). The total puncta density was not affected by zinc application (Supplementary Figure S5B), which agrees with the stable mEPSCs frequency during zinc treatment (Figure 4F). The relative rate between exocytosis and lateral diffusion was likely not affected by zinc since there was no difference in the slope of Alexa 647-CAM2 to Alexa 488-CAM2 (Supplementary Figure S5C).

Next, to assess whether there is a direct correlation between the amount of Shank2 or Shank3 and Alexa-labeled CAM2, we measured the fluorescence intensity of Shank at each individual puncta as well as the intensity of the CAM2 in both channels. Fluorescence intensities of individual Alexa-labeled CAM2 puncta were then plotted as a function of the corresponding colocalized Shank2 or Shank3 puncta intensities, and linear regression analysis applied for the individual synaptic intensities (Figures 9E–H and Supplementary Figures S5D,E O’Brien et al., 1998; Rumbaugh et al., 2003). Consistent with previous results (Figures 8B,C), we found a significant increase in the slope of A647-CAM2 intensity at Shank3-positive puncta with zinc treatment (Figures 9E,G), which means that for a given amount of Shank3 there is an increase in A647-CAM2. This can serve as a proxy for the increase in ratio of GluA2 to Shank3 through lateral diffusion (A647-CAM2; 139.8%, *p* = 0.0001; Figure 9G) or through exocytosis (A488-CAM2; 48.66%, *p* = 0.0186; Figure 9H). In contrast, the correlation between Shank2 and GluA2 for both processes was unchanged with zinc treatment (Figures 5G,H and Supplementary Figures S5D,E), implying that Shank2 did not directly influence the zinc-sensitive dynamics of GluA2. Taken together, these results imply that: (1) both lateral diffusion and exocytosis contributed to the GluA2 pool recruited by zinc; and (2) Shank3 was involved in both processes.

### Characterization of Shank2 and Shank3 Knockdown in Young Neurons

**Figure 10:**
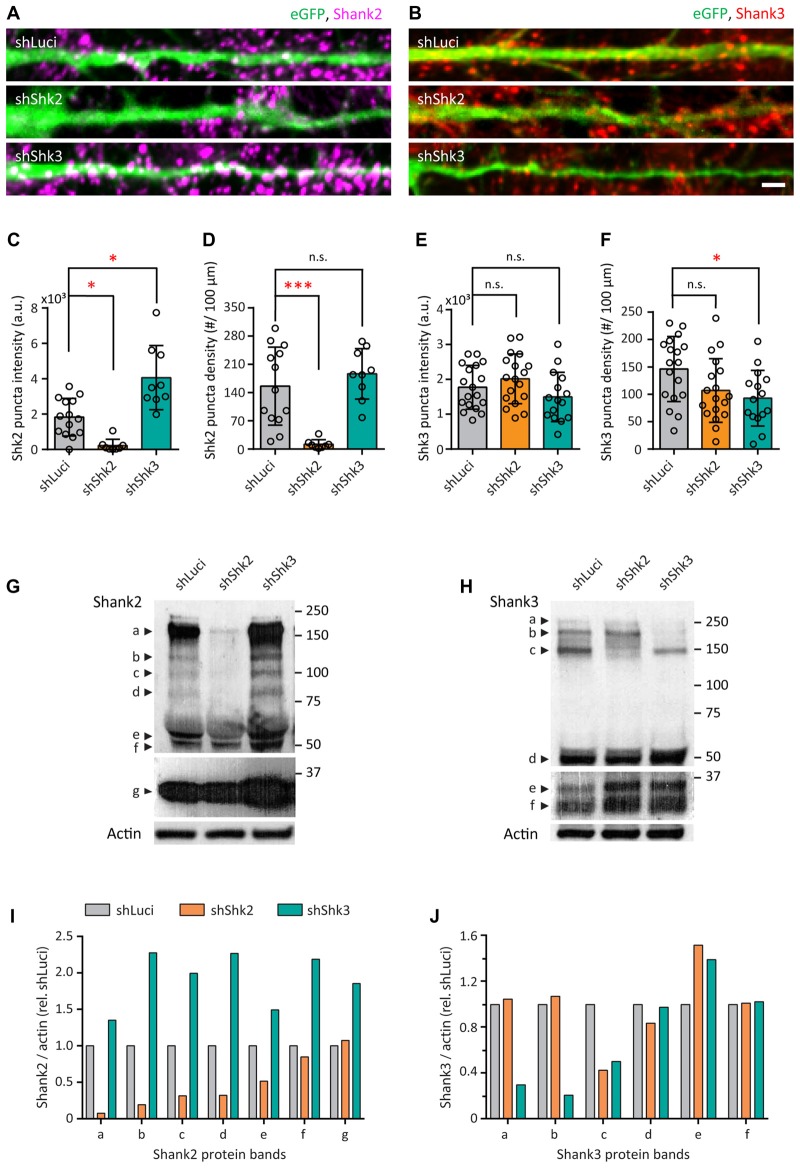
Short-hairpin RNA (shRNA)-mediated knockdown of Shank2 and Shank3. **(A,B)** Straightened dendrites of transfected hippocampal neurons expressing a bicistronic construct with enhanced green fluorescent protein (eGFP; green) and shRNA hairpins for shLuciferase (shLuci, top), shShank2 (shShk2, middle) or shShank3 (shShk3, bottom) and immunostained for Shank2 (magenta; **A**) or Shank3 (red; **B**) after 14 DIV. Scale bar: 5 μm.** (C–F)** Summary graphs showing quantification of Shank2 **(C,D)** and Shank3** (E,F)** puncta intensity and density (mean ± SEM) for different knockdown conditions (Kruskal-Wallis one-way ANOVA with Dunn’s correction *post hoc* multiple comparisons, Shank2 staining: shLuci, *N* = 14; shShk2, *N* = 8; shShk3, *N* = 9 dendrites from 8 to 12 neurons from three culture preps. Shank3 staining: shLuci, *N* = 18; shShk2, *N* = 18; shShk3, *N* = 15 dendrites from 8 to 12 neurons from three culture preps; n.s. *p* ≥ 0.05, *p < 0.05, ***p < 0.001).**(G,H)** Western blots of cellular lysates from dissociated hippocampal neurons infected with lentivirus for shLuci, shShk2 or shShk3 immunoblotted for Shank2 **(G)** or Shank3 **(H)** antibodies. Protein molecular weights are indicated at the right in kDa. Different isoforms are labeled at the left. Top two panels in **(G,H)** show different exposures of the same films for optimal visualization of different isoforms. Actin was used as a loading control (bottom panels in **G,H**). **(I,J)** Quantification of Shank2 **(I)** or Shank3 **(J)** protein levels for different isoforms in hippocampal neurons for different knockdown conditions from two cultures per condition. “Shank/actin (rel. shLuci)” refers to the expression level of Shank2 or Shank3 normalized to actin and then to shLuci control.

To understand if Shank2 or Shank3 are required for the zinc-sensitive regulation of AMPAR structure and functions, we employed shRNA to decrease the endogenous expression of Shank2 or Shank3 in neurons (Figure 10). Multiple shRNA constructs for Shank2 and Shank3 were designed in a pZoff vector to target the various isoforms of each protein (Supplementary Figures S6A,B and Supplementary Tables S1, S2; Boeckers et al., 1999; Lim et al., 1999; Leal-Ortiz et al., 2008). To assess their efficacy in neurons, we used plasmid-based transfection and immunostained for Shank2 and/or Shank3 (Figures 10A,B and Supplementary Figure S6C). shRNA-Shank2 (shShk2) targeting the proline-rich domain of Shank2 (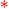 in Supplementary Figure S6A) was the most effective, reducing Shank2 puncta intensity by 87.5% (*p* = 0.0335; Figure 10C) and decreasing the puncta density by 90.56% (*p* = 0.001; Figure 10D). The most effective shRNA for Shank3 targeting the 3′UTR domain (shShk3; 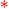 in Supplementary Figure S6B) showed a significant decrease in Shank3 puncta density (35.56%, *p* = 0.0372; Figure 10F) and no change in intensity (Figure 10E), accompanied by a 122.08% increase of Shank2 puncta intensity (*p* = 0.0233; Figure 10C).

We next subcloned the two successful shRNAs described above into a lentivirus vector to create LV/eGFP/shRNA constructs (Supplementary Figure S6D) to ensure higher infection efficiency and avoid any potential overexpression artifacts associated with plasmid-based transfections. Lysates of hippocampal neurons infected with lentiviruses at 100% infectivity were harvested after 14 DIV and probed with antibodies for Shank2 and Shank3 (Figures 10G,H and Supplementary Figures S6E,F). With the shShk2, we observed a dramatic loss of most major Shank2 isoforms (Figure 10I) along with the reduction of the third longest isoform of Shank3 (c: 58.98% Figure 10J). The effect of shShk2 on Shank3 expression was similar to previous studies suggesting that Shank2 might be necessary for recruiting synaptic Shank3 over development (Grabrucker et al., 2011; Shi et al., 2017). On the other hand, the shShk3 produced ~50%–80% loss of the three longest Shank3 isoforms (a: 70.05%, b: 80.01%, c: 53.6%; Figure 10J), which are the major zinc-binding isoforms. This shRNA also induced large increases of all Shank2 isoforms (Figure 10I), suggesting that there might be a compensation of Shank2 due to the loss of Shank3.

**Figure 11:**
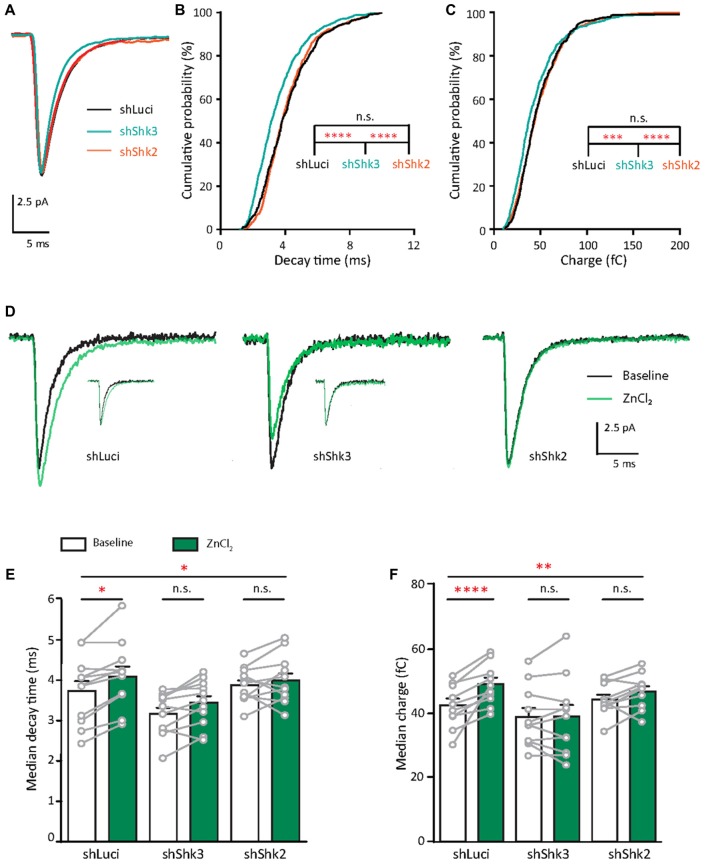
Shank knockdown alters AMPAR function and suppresses their zinc-dependent changes. **(A)** Ensemble-averaged mEPSCs from recordings of hippocampal neurons infected with lentiviruses expressing shLuci (black), shShk3 (blue) or shShk2 (orange; *N* = 11 cells per condition from five to seven culture preps per condition).** (B,C)** Cumulative probability histograms of decay time **(B)** and charge **(C)** of isolated events of different conditions (Kruskal-Wallis one-way ANOVA followed by Dunn’s correction *post hoc* multiple comparisons, *N* = 400–600 events from 11 neurons from five to seven culture preps per condition; n.s. *p* ≥ 0.05, ***p < 0.001, ****p < 0.0001). **(D)** Average mEPSCs from recordings at baseline (black) and zinc (green) conditions of individual hippocampal neurons infected with lentiviruses expressing shLuci (left), shShk3 (middle) or shShk2 (right; *N* = 11 cells from five to seven culture preps per condition). mEPSCs normalized to baseline response are shown in corresponding insets to differentiate effect of zinc on decay kinetic. **(E,F)** Summary graphs with per-cell-basis pairwise comparisons of decay time **(E)** and charge **(F)** between baseline and ZnCl_2_ conditions in different conditions (median ± SEM; two-way ANOVA; *p < 0.05, **p < 0.01. Sidak correction multiple *post hoc* comparisons, *N* = 10–11 cells from five to seven culture preps per condition; n.s. *p* ≥ 0.05, *p < 0.05, ****p < 0.0001).

Next, we assessed the effects of Shank2 and Shank3 knockdown on AMPAR function. Here, we compared AMPAR-mediated mEPSCs from neurons infected with shLuci (control), shShk2 or shShk3 (Figure 11A). Knockdown of Shank2 led to a modest reduction of mEPSCs amplitude as seen in the cumulative distribution (5.18%, *p* = 0.0072; Supplementary Figure S7A). In contrast, the AMPAR response in Shank3 knockdown neurons had a faster rise time (9.6%, *p* = 0.0032; Supplementary Figure S7B) and decay time (20.89%, *p* < 0.0001; Figure 11B). The faster kinetics led to a reduction in charge transferred by AMPAR in Shank3 knockdown neurons (16%, *p* = 0.0004, Figure 11C). Taken together, these results support that Shank3 is important for maintaining the kinetics, and hence, synaptic efficacy of AMPAR synaptic response in young neurons.

### Zinc-Sensitive Regulation of AMPAR in Young Neurons Is Dependent on Shank2 and Shank3

Given that 10 μM zinc treatment led to increased incorporation of GluA2 to Shank puncta (Figures 8, 9), we next asked whether Shank expression is necessary for the zinc-sensitive increase of AMPAR function. To address this question, we recorded and compared AMPAR mEPSCs from shLuci, shShk3 and shShk2 neurons at baseline and during zinc treatment (Figure 11D). Neurons infected with shLuci preserved their zinc-sensitivity with an increase in decay time (12.63%, *p* = 0.0069) and peak amplitude (30.44%, *p* = 0.044; Figure 11E and Supplementary Figure S7C). As a result, the charge transferred by AMPARs in shLuci neurons was increased by zinc treatment (16%, *p* < 0.0001; Figure 11F). The effects of zinc on shLuci neurons are comparable to those on untransfected neurons described in Figure 4 (*p* > 0.05).

In contrast, we observed no zinc-sensitive increase in either decay time or charge with zinc addition in shShK3 neurons, confirming that synaptic expression of Shank3 is necessary for the zinc recruitment of GluA2-containing AMPARs (Figures 11E,F). Instead of being potentiated by zinc treatment, mEPSCs in Shank3 knockdown neurons displayed a decrease in peak amplitude (37.42%, *p* = 0.012) and frequency (34.78%, *p* = 0.035) upon zinc treatment (Supplementary Figures S7C,E). This observation revealed a separate zinc-dependent modulation of AMPAR activity, perhaps via the reduction of surface GluA1 as described in Figure 7 from Shank synapses. Furthermore, knockdown of Shank2 also abolished the zinc sensitivity of AMPARs in all measures examined (decay time, charge, amplitude and rise time; Figures 11E,F and Supplementary Figures S7C–E). Together, these data revealed the necessity of both Shank2 and Shank3 expression to zinc-induced potentiation of AMPAR function.

## Discussion

The dynamic regulation of neurotransmitter receptor number and composition is a fundamental mechanism underlying synaptic plasticity, synaptic maturation and neural circuit development (Henley et al., 2011; Bassani et al., 2013; Henley and Wilkinson, 2016). This regulation is critical for the encoding of information, cognition and behavior and is vulnerable to genetic and environmental insults associated with ASD (Shepherd and Huganir, 2007; Lee et al., 2016; Kim et al., 2018). In this study, we explored the molecular mechanisms underlying zinc-dependent regulation of synaptic transmission via the postsynaptic scaffolding proteins Shank2 and Shank3. Our data reveal that young hippocampal neurons undergo a zinc-dependent subunit switch of AMPAR from GluA2-lacking to GluA2-containing receptors, which dictate their biophysical properties. In addition, we found that Shank proteins are key mediators of this regulation since they were necessary for this zinc-induced enhancement of AMPARs. Importantly, postsynaptic zinc, an activator of Shank2 and Shank3, was found to increase transiently and reversibly with neuronal depolarization, likely due to release of zinc from presynaptic vesicles or postsynaptic sources. Upon treatment with zinc, GluA2 was preferentially recruited into synapses by both lateral diffusion and exocytosis with a concomitant dispersion of GluA1. This occurred at pre-existing Shank2 and Shank3 puncta in young neurons, converting these synapses from GluA2-lacking to GluA2-containing. This zinc-stimulated subunit switch of surface GluA2 was accompanied by an increase in amplitude, longer decay time and reduced inward rectification of AMPAR-mediated currents and was dependent on Shank2 and Shank3. In summary, these results provide new insights into a cooperative dynamic regulation of AMPAR composition driven by the zinc signaling pathway via Shank2 and Shank3 at developing synapses, a critical period for local control of AMPAR composition.

### Bimodal Action of Zinc on Shank2 and Shank3 for Dynamic Regulation of AMPA Receptors

During development and plasticity, GluA2 is a tightly regulated subunit of glutamate receptors (Isaac et al., 2007). GluA2-containing AMPARs at synapses are controlled through a variety of mechanisms and significantly increase with maturation (Pickard et al., 2000; Kumar et al., 2002; Brill and Huguenard, 2008; Mignogna et al., 2015). Here, we found GluA2 was recruited to an increasing number of synapses in dissociated hippocampal neurons, almost doubling during our developmental window of interest (Figure 2). Shank3 likewise increased in expression at individual synapses and number of synapses in this same period with a particular recruitment to mature synapses on dendritic spines (Figure 1). The SH3 domain of Shank proteins binds to GRIP suggesting that Shank3 might indirectly interact with and recruit GluA2 to synapses via the APB/GRIP complex (Sheng and Kim, 2000). Functionally, the knockdown of Shank3 has been shown to result in decreased GluA2 expression and GluA2-mediated AMPAR properties, such as reduced inward rectification (Bariselli et al., 2016; Mei et al., 2016). In our current study, Shank3 knockdown resulted in AMPAR mEPSCs with a faster decay time (Figure 11), which supports the concept that Shank3 can promote synaptic clusters of GluA2. Furthermore, Shank3 was shown to directly facilitate GluA2-containing AMPAR activity since zinc preferentially enhanced GluA2 at Shank3 puncta (Figure 8 and Table 1). Both Shank2 and Shank3 appear to be necessary for the zinc-induced enhancement of synaptic AMPARs since knocking down either Shank3 or Shank2 eliminated the zinc effect. In both cases, this is likely due to a reduction of Shank3 at synapses (Figure 10), consistent with a recent study showing Shank2 is necessary for recruitment of Shank3 (Shi et al., 2017).

Clues to the mechanism for how Shank3 and zinc recruit GluA2-containing AMPAR to synapses can be found in the role of Shank3 in receptor trafficking (Okamoto et al., 2001; Lu et al., 2007; Verpelli et al., 2011; Raynaud et al., 2013), and zinc may be a key underlying driver. For example, zinc could facilitate the interaction between Shank3 and the Homer1b/dynamin-3 complex to tether the endocytic zone in close proximity to the PSD. This would allow for more rapid receptor recycling that could increase total surface GluA2 (Okamoto et al., 2001; Lu et al., 2007; Petrini et al., 2009). Using CAM2 labeling, we showed that the magnitude of the zinc-induced recruitment of GluA2 via lateral diffusion and exocytosis was correlated to the amount of synaptic Shank3 (Figure 9). Additionally, we found that elevating zinc induced a trafficking of GluA2 to Shank3-positive puncta from non-Shank3 sites (Figure 8 and Table 1). These functions are plausible, especially considering the strong expression level of Shank3 in dendrites and its known interaction with the cytoskeleton via cortactin/actin binding (MacGillavry et al., 2016).

Since zinc-mediated recruitment of GluA2-containing AMPAR to Shank synapses did not strongly affect amplitude or frequency for AMPA mEPSCs, this implies that zinc might concomitantly induce the dispersion/removal of other types of synaptic AMPARs. Indeed, our data revealed that elevation of zinc also induced a synaptic removal of GluA1 from Shank2-positive synapses, indicating that GluA1 dispersion may be mediated by Shank2 (Figures 8, 11). Consistent with this concept, previous studies showed that Shank2 directly associates and colocalizes with GluA1 and is critical for its synaptic expression, particularly at nascent synapses (Pickard et al., 2000; Uchino et al., 2006; Ha et al., 2016; Peter et al., 2016; Szíber et al., 2017). These findings indicate that Shank2 may regulate the synaptic localization of GluA1. In support of this idea, our data confirmed that Shank2 initially co-clustered with surface GluA1 (Figure 8 and Table 1) and knockdown of Shank2 led to reduction of AMPAR mEPSC amplitude (Supplementary Figure S7) in young neurons, potentially indicative of fewer GluA1 receptors. Furthermore, Shank2 overexpression, a condition induced by expressing shShk3, also resulted in synapses with faster kinetics (Supplementary Figure S7B and Figures 11B,C) perhaps via the increase recruitment of GluA1 to Shank2 sites. In contrast, elevating zinc reduced surface GluA1 puncta density. This could be due to increased endocytosis and/or diffusion of GluA1 away from Shank2-positive sites (Figure 8 and Table 1). In line with a role of Shank2 and zinc in the synaptic removal of GluA1, our data showed that the zinc-induced reduction of AMPAR mEPSCs amplitude was prominent at synapses lacking Shank3 (Supplementary Figure S7C), a condition that creates synapses dominated by Shank2 (Figure 10). This effect of zinc, however, failed to occur in Shank2-lacking synapses (Supplementary Figure S7C).

Several mechanisms could explain the effects of zinc on downregulating GluA1 level at Shank2 sites. First, zinc binding to the SAM domain could favor a new conformation of Shank2 within the Shank scaffolding network or the complex with other molecules, all of which could have lower affinity or fewer docking sites for GluA1. This potential conformational change of Shank2 might affect its interaction with dynamin-2 (Okamoto et al., 2001) to facilitate GluA1 internalization (Carroll et al., 1999). Additionally, zinc could affect GluA1 synaptic dispersion by regulating the PKA, PKC or CaMKII-dependent phosphorylation of this AMPAR subunit either directly (Noh et al., 2001) or via crosstalk with calcium signaling (Hershfinkel et al., 2001; Takeda et al., 2008). Intriguingly, reduced phosphorylation of GluA1 at sites S831 and S845 has been reported in a Shank2 knockout mouse model (Won et al., 2012), raising yet another possible mechanism for zinc to regulate Shank2-dependent redistribution of GluA1.

**Figure 12:**
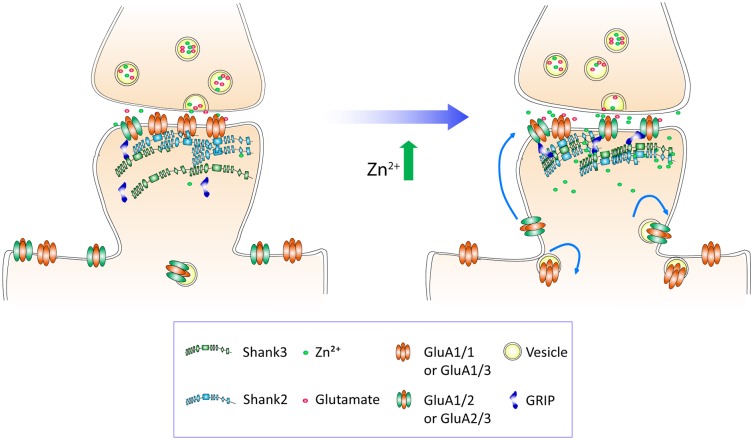
A model for Shank mediation of zinc-dependent subunit switch of AMPAR in developing synapses. A glutamatergic synapse of a wild-type (WT) young neuron is shown with low levels of Shank3 (green) and GluA2 (teal) and high levels of Shank2 (blue) and GluA1 (orange). The synaptic complement changes with zinc-sensitive recruitment of synaptic GluA2 via exocytosis and lateral diffusion (blue arrows), leading to potentiation of AMPAR-mediated synaptic activity. The zinc-recruitment of GluA2 correlated with the level of synaptic Shank3. Zinc addition also led to reduction of synaptic GluA1, which is most likely driven by Shank2. Hence, loss of either Shank2 or Shank3 abolished the zinc-sensitive potentiation of AMPAR-mediated synaptic transmission.

In summary, the data presented in this study suggest a model for how zinc and zinc-sensitive Shank proteins regulate AMPAR function at developing synapses. Under basal conditions in young neurons, Shank2 appears to promote the synaptic localization and molecular anchoring of GluA1-containing receptors. Elevating zinc near synapses, as occurs during synaptic transmission, then exerts two effects: preferentially recruiting GluA2 to Shank3 synapses through both lateral diffusion and exocytosis, while simultanously promoting the removal of GluA1 receptors from Shank2 complexes (Figure 12). In this model, zinc interactions with Shank molecules may offer a general mechanism to shape the biophysical properties at developing glutamatergic synapses. While there are clear differences in the effects of shShk2 and shShk3 on Shank2 and Shank3 expression, both shRNAs induced changes in protein levels of these molecules. Future studies should attempt to parse the specific roles of Shank2 and Shank3 using strategies that result in cleaner knockdown or knockout of each protein.

### Functional Implications of Zinc-Sensitive Control of AMPAR Subunit Composition

Our results suggest that a major function of the synaptic zinc-Shank pathway is to facilitate synaptic maturation. Shank3 was found to be necessary for AMPAR maturation in the ventral tegmental area (Bariselli et al., 2016). It is likely that during development of hippocampal neurons, Shank2 and Shank3 act as zinc-sensitive mediators to convert GluA2-lacking to GluA2-containing AMPAR synapses. More specifically, our data support the proposed model in which zinc could trigger Shank3 to accumulate more GluA2-containing AMPAR and act on Shank2 to remove GluA1-containing AMPAR over time. Perturbations of this pathway specifically during the prenatal period or early postnatal development have been linked to delayed AMPAR maturation, circuit dysfunction and behavioral deficits, further emphasizing the role of zinc-sensitive Shank proteins and zinc in synapse maturation and for the formation of associated brain circuits (Peça et al., 2011; Grabrucker et al., 2014; Bariselli et al., 2016; Mei et al., 2016). It is intriguing to speculate that other features of synaptic maturation, such as the subunit switch of NMDAR, might also be mediated by zinc and zinc-sensitive Shank proteins and that these processes might be shared mechanisms for synaptic development in pyramidal neurons of other brain areas. In order to directly understand whether zinc signaling mediates synaptic maturation, future studies could focus on revealing the effect of chronic chelation of zinc in the transition period from young to mature neurons during the development of synapses. However, a major challenge of such an approach is to select the appropriate zinc chelator that does not severely impair neuronal health, synapse stabilization or synaptic localization of Shank2 and Shank3 (Grabrucker et al., 2011; Arons et al., 2016). Moreover, since the source of zinc to activate Shank2 and Shank3 could be from either presynaptic vesicles or postsynaptic stores, both extracellular and intracellular zinc chelators should be considered to selectively chelate presynaptic- or postsynaptic-released zinc, respectively (Besser et al., 2009; Pan et al., 2011; Grabrucker et al., 2014).

The loss of AMPAR zinc-sensitivity in mature neurons (Figure 6) might be regulated by a variety of mechanisms. During early stages of development, the size of the synapse, and the number and composition of receptors with their complement of proteins are plastic (Craig et al., 1993; Rao et al., 1998; Boeckers et al., 1999; Kumar et al., 2002; Grabrucker et al., 2011; Valnegri et al., 2011). In the presence of zinc and zinc-sensitive Shank molecules, synapses are more dynamic and less stable (Grabrucker et al., 2011; Arons et al., 2012, 2016). Additionally, young synapses could have more available docking sites to accommodate zinc-induced recruitment of GluA2-containing receptors (Czöndör et al., 2012). Over development, the increase of Shank1 at synapses (Grabrucker et al., 2011) and the loss of zinc-sensitive recruitment of molecules may synergistically create a more stable/less plastic state. Also, a reduction in the diffusion rate of AMPAR could hinder zinc-sensitive recruitment of AMPAR via lateral diffusion in mature neurons (Groc et al., 2006; Czöndör et al., 2012). Another possibility is that the high content of GluA2 in mature neurons (Figure 2) could result in the reduction of AMPAR zinc sensitivity (Figure 5D) by affecting zinc entry (Jia et al., 2002; Takeda et al., 2007). However, we found that intracellular zinc levels of neurons at different DIV (11–30) were similarly responsive to exogenous manipulations (Supplementary Figure S2), suggesting that the availability of zinc is not a limiting factor for mature neurons to respond to treatment. The mechanism shown here could allow for a means to downregulate AMPAR calcium signaling as seen in mature neurons. Considering that mature neurons can uptake zinc, it is possible a smaller subpopulation of synapses in mature neurons could undergo the AMPAR subunit switch dependent on their experience (Mattison et al., 2014). Furthermore, mature neurons may have other zinc-sensitive processes that play alternate roles in neuronal function, such as plasticity.

Does the zinc-sensitive dynamic control of AMPAR composition operate during plasticity? While zinc and zinc-sensitive Shank proteins may act together to regulate maturation of synapses, it is also reasonable to consider that they may additionally function to modulate AMPAR subunit composition during synaptic plasticity, e.g., during the induction and maintenance of long-term potentiation (LTP). More specifically, activity-dependent accumulation of postsynaptic zinc might activate Shank2 and Shank3 to replace GluA1-containing AMPARs with GluA2-containing receptors (Shi et al., 2001; Alberi et al., 2005). This AMPAR subunit switch mechanism might also enable synaptic long-term depression (LTD), especially at newly unsilenced synapses (Zhou et al., 2011; Selcher et al., 2012). Hence, the differential zinc sensitivity of synapses could allow them to be dynamically remodeled during Hebbian plasticity. Conversely, dysfunction of this pathway could contribute to the synaptic plasticity deficits seen in multiple Shank knockdown or zinc-deficient models (Lu et al., 2000; Jiang et al., 2011; Verpelli et al., 2011; Wang et al., 2011, 2016; Jaramillo et al., 2016). In another aspect of plasticity, a bidirectional model for synaptic scaling has emerged in which scaling up is dependent on increased synaptic accumulation of GluA2-containing receptors (Gainey et al., 2009; Anggono et al., 2011; Tatavarty et al., 2013; Ancona Esselmann et al., 2017). During synaptic scaling up due to chronic activity deprivation, one trafficking mechanism was shown in which GRIP was recruited to synaptic sites where it enhanced trafficking and/or stabilization of GluA2 (Gainey et al., 2015; Tan et al., 2015). Since Shank proteins have been reported to bind to GRIP and therefore could complex with GluA2 through the APB/GRIP (Sheng and Kim, 2000; Uemura et al., 2004), the zinc-sensitive dynamic regulation of AMPAR at developing synapses may converge in this same pathway. In general, this zinc-sensitive regulation of AMPAR could play roles to modulate receptor compositions and synaptic strength.

### Zinc, Zinc-Sensitive Shank Proteins and Autism Spectrum Disorders

Our study revealed a novel zinc/Shank-dependent molecular pathway for regulating AMPAR subunit switching that could affect the formation, maturation and plasticity of excitatory synapses. Consistently, alterations of AMPAR subunit compositions and/or synaptic recruitment have been seen in multiple Shank2 and Shank3 mouse KO models of ASDs (Ramanathan et al., 2004; Mejias et al., 2011; Hayashi et al., 2013; Mignogna et al., 2015; Bariselli et al., 2016; Chanda et al., 2016; Wegener et al., 2017). Our findings also offer a novel mechanism for understanding how zinc deficiency or disrupted zinc dynamics might be linked to individuals with ASDs (Yasuda et al., 2011; Grabrucker et al., 2014; Curtin et al., 2018). Specifically, we anticipate that zinc deficiency during early brain development could disrupt Shank functions (Grabrucker et al., 2011; Grabrucker, 2014), the composition of synaptic AMPAR and ultimately synaptic plasticity, network formation and behavior. With shared molecular deficits to Shank knockout mice, zinc deficiency could result in similar circuit and behavioral deficits broadly in a number of relevant animal models (Halas and Sandstead, 1975; Sandstead et al., 1978; Lu et al., 2000; Grabrucker et al., 2014; Hagmeyer et al., 2015). Taken together, our study adds to the current understanding of how Shank2 and Shank3 regulate multiple aspects of synaptic functions during development and plasticity. These results also indicate that genetic mutations and environmental insults might predispose individuals to ASDs by impairing this process. This realization might help improve future diagnostics and development of effective pharmacotherapies for ASDs (Grabrucker et al., 2014; Lee J. et al., 2015; Bariselli et al., 2016; Hagmeyer et al., 2018).

## Author Contributions

CG, JM and SAK conceived and developed the initial concepts for the project. HH, CG, JH and SAK designed the research. HH and SAK performed all experiments and wrote the manuscript in consultation with CG and JH. SL-O helped design shRNA and provided technical input for cell culture and biochemistry experiments. IH and SK provided CAM2 reagents and technical consultation for CAM2 experiments. HH, KL and SAK analyzed the data. SPM wrote the IMFLAN3D and SpineZap analysis packages and gave technical support for image analysis. All authors revised the manuscript.

## Conflict of Interest Statement

The authors declare that the research was conducted in the absence of any commercial or financial relationships that could be construed as a potential conflict of interest.

## Footnotes

^1^https://hlab.stanford.edu/wdetecta.php/
