# Supplementary material for: Shank and Zinc Mediate an AMPA Receptor Subunit Switch in Developing Neurons
Source: Front Mol Neurosci. 2018 Nov 9;11:405. doi: 10.3389/fnmol.2018.00405 (PMC6256285; doi:10.3389/fnmol.2018.00405)
Supplement: Supplementary file 1 [file Presentation_1.pdf]

Supplementary Material

Shank and zinc mediate an AMPA receptor subunit switch in developing neurons

Huong T. T. Ha, Sergio Leal-Ortiz, Kriti Lalwani, Shigeki Kiyonaka, Itaru Hamachi, Shreesh P. Mysore, Johanna M. Montgomery, Craig C. Garner*, John R. Huguenard*, Sally A. Kim*

*** Correspondence:**

Sally A. Kim: sallykim@stanford.edu

John R. Huguenard: huguenar@stanford.edu

Craig C. Garner: craig-curtis.garner@dzne.de

Supplementary Figure S1

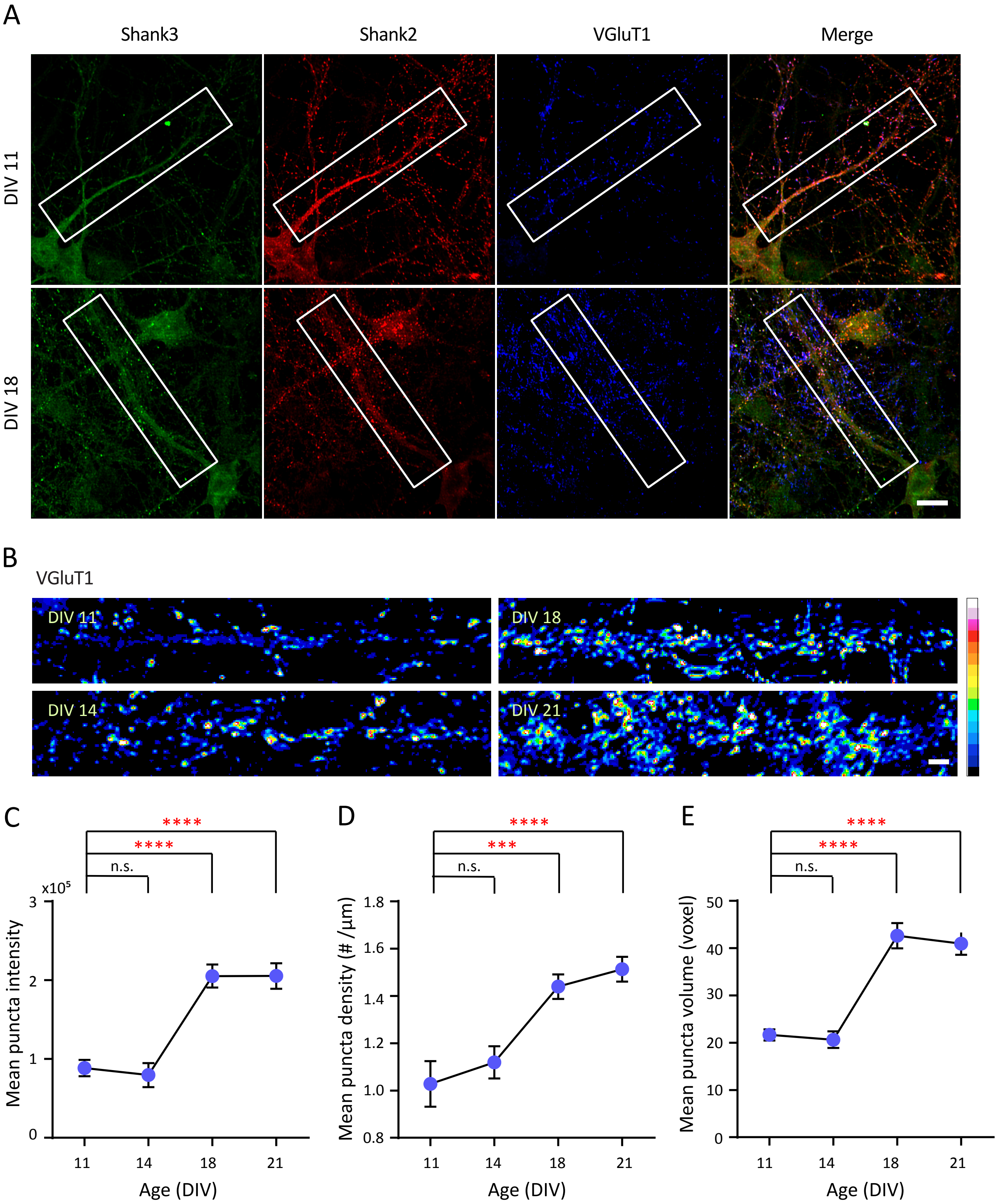

Supplementary Figure S1 | VGluT1 expression increases during development. (A) Young (DIV 11) and mature (DIV 18) hippocampal neurons co-immunostained for Shank3 (A488, green), Shank2 (A568, red) and VGluT1 (A647, blue). White puncta in the merge images (far right) indicate colocalization of all three proteins. Scale bar: 20 µm. White boxes mark dendrites shown in **Figure 1**. **(B)** Dendrites of young (DIV 11 & 14) and mature (DIV 18 & 21) hippocampal neurons immunostained for VGluT1. Scale bar: 5 µm. **(C-E)** Three-dimensional analysis of VGluT1 puncta at DIV 11, 14, 18 and 21 quantifying intensity **(C)**, density **(D)** or volume **(E)** (mean ± SEM; DIV 21, N = 18; all others, N = 16 from 10-12 neurons from 2 culture preps; Kruskal-Wallis one-way ANOVA followed by Dunn's correction post-hoc multiple comparisons. n.s. $p \geq 0.05$, *** $p < 0.0005$, **** $p < 0.0001$).

Supplementary Figure S2

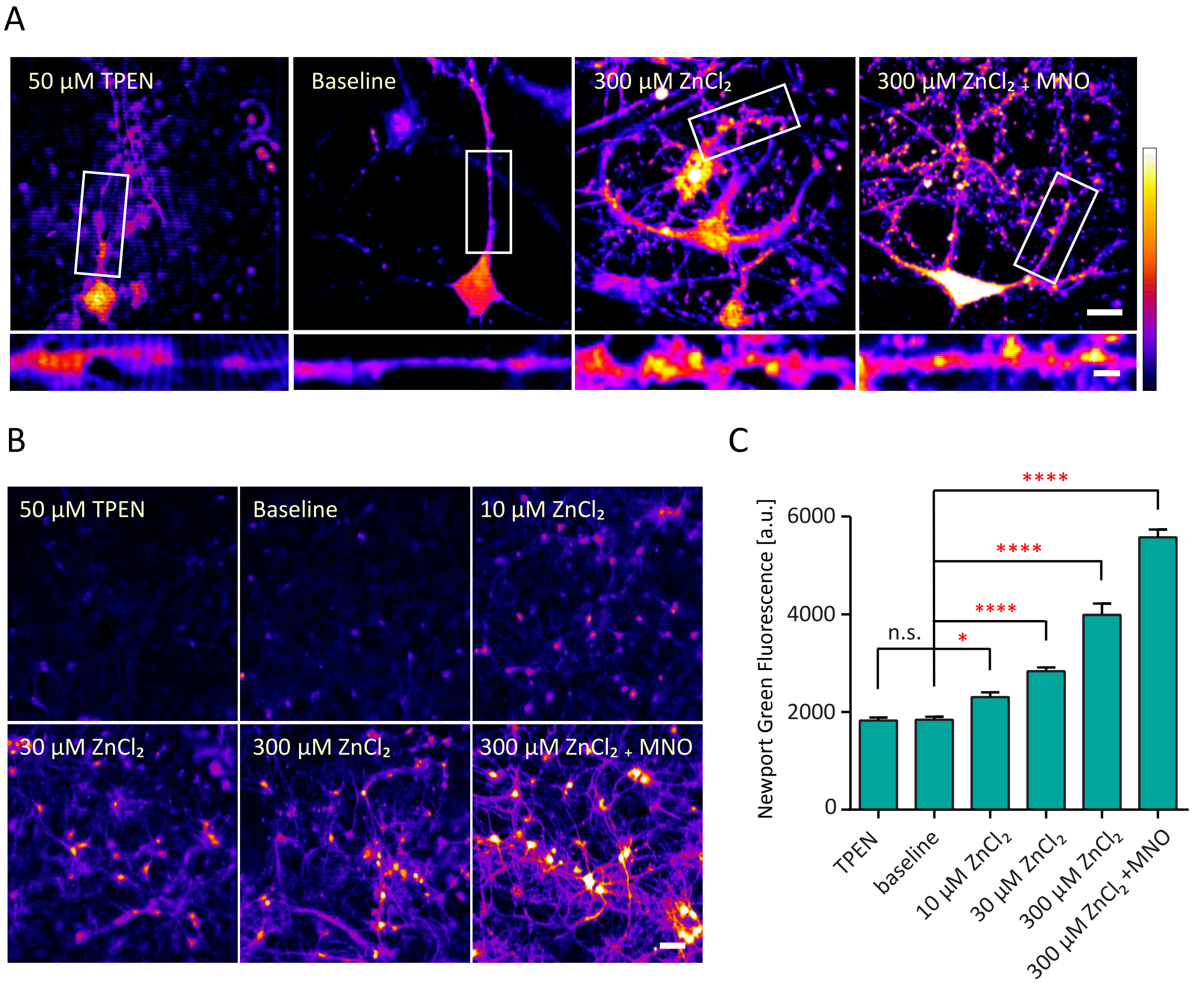

Supplementary Figure S2 | Exogenous application of $ZnCl_2$ elevates intracellular zinc concentration. (A) Hippocampal neurons (top) were loaded with Newport Green DCF (NPG) and treated with different concentrations of $ZnCl_2$ [± pyrithione (MNO)] or TPEN as specified. White boxes mark the straightened dendrites shown in the lower panels. Scale bar: Image, 10 µm; Corresponding inset, 5 µm. **(B)** Populations of hippocampal neurons were labeled with NPG and treated with different conditions of $ZnCl_2$ (± MNO) or TPEN as indicated. Scale bar: 50 µm. **(C)** Effects of extracellular TPEN or $ZnCl_2$ (± MNO) applications on somatic NPG. [Kruskal-Wallis one-way ANOVA followed by Dunn's correction multiple comparisons. N (cell somas from 4-6 coverslips from three culture preps): 230 (50 µM TPEN), 253 (baseline), 109 (10 µM $ZnCl_2$), 207 (30 µM $ZnCl_2$), 103 (300 µM $ZnCl_2$) and 203 (300 µM $ZnCl_2$ + MNO). n.s. $p > 0.05$, * $p < 0.05$, **** $p < 0.0001$].

Supplementary Figure S3

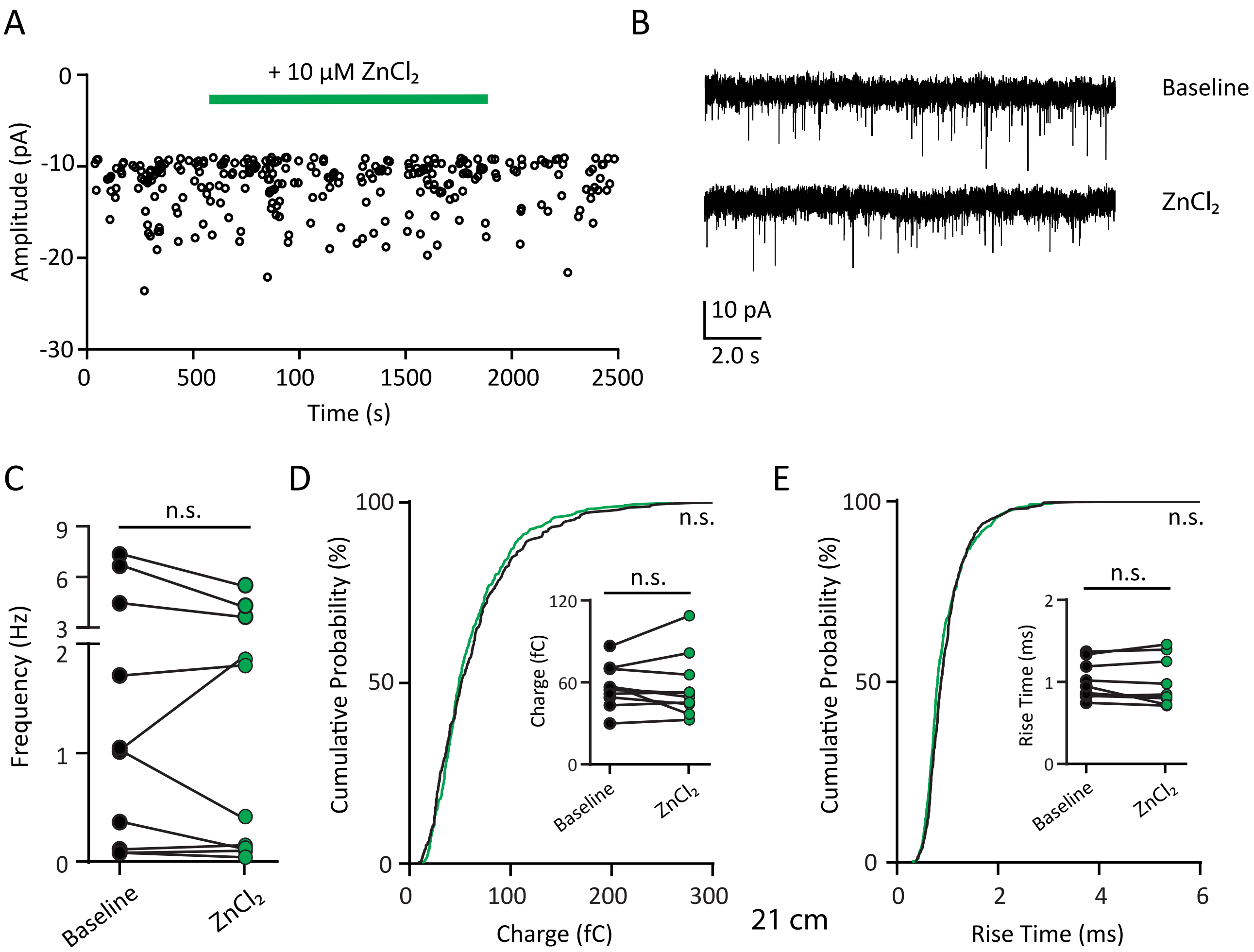

Supplementary Figure S3 | Zinc treatment does not affect AMPAR mEPSCs in mature neurons. (A) AMPAR mEPSCs were recorded from a mature hippocampal neuron. Green bar: 10 µM $ZnCl_2$ application. **(B)** AMPAR mEPSCs recording traces from an individual neuron at baseline (top) and during $ZnCl_2$ (bottom). **(C)** Pairwise comparison of median AMPAR mEPSC frequency between baseline and $ZnCl_2$ conditions. **(D-E)** Cumulative probability histograms of charge **(D)** or rise time **(E)** of isolated events from baseline (black) and $ZnCl_2$ (green) conditions (K.S. test, N = 400 events per condition. n.s. $p \geq 0.005$). Insets display per-cell-basis pairwise comparison of charge **(D)** or rise time **(E)** between baseline and $ZnCl_2$ conditions. (Wilcoxon test, N = 10 cells from 3 culture preps. n.s. $p \geq 0.05$).

Supplementary Figure S4

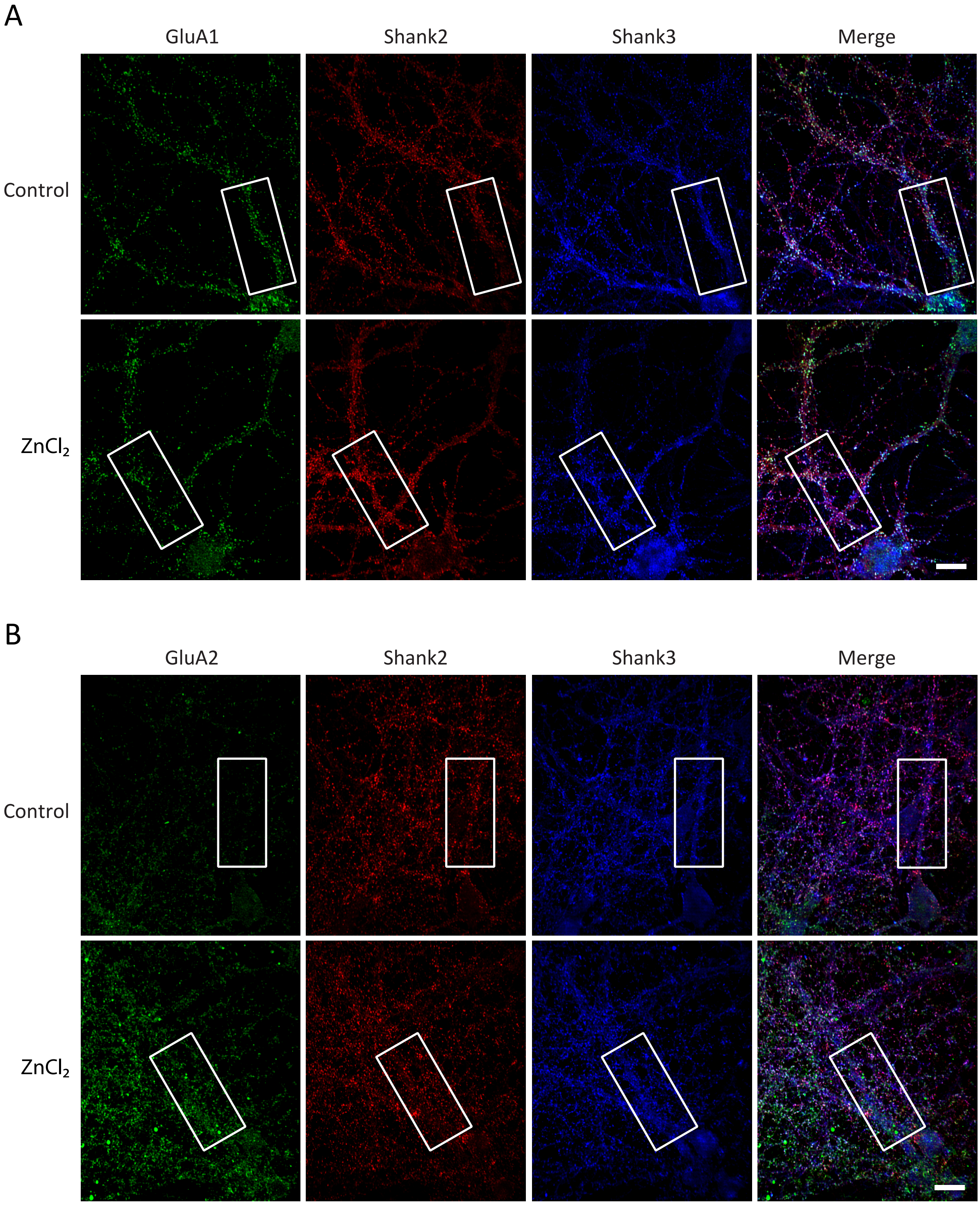

Supplementary Figure S4 | Zinc treatment alters the colocalization between AMPAR subunits and Shank proteins. (A) Young hippocampal neurons (DIV 14) treated with control (top) or 10 μM $ZnCl_2$ (bottom) conditions. Neurons were live surface labeled for GluA1 (A488, green, left) before being fixed and co-immunostained with antibodies for Shank2 (A568, red, second from the left) and Shank3 (A647, blue, third from the left). White puncta in the merge images (far right) indicate colocalization of all three proteins. White boxes mark dendrites shown in **Figure 8**. Scale bar: 20 μm. **(B)** Similar to **(A)**, but neurons were live labeled with GluA2 instead of GluA1.

Supplementary Figure S5

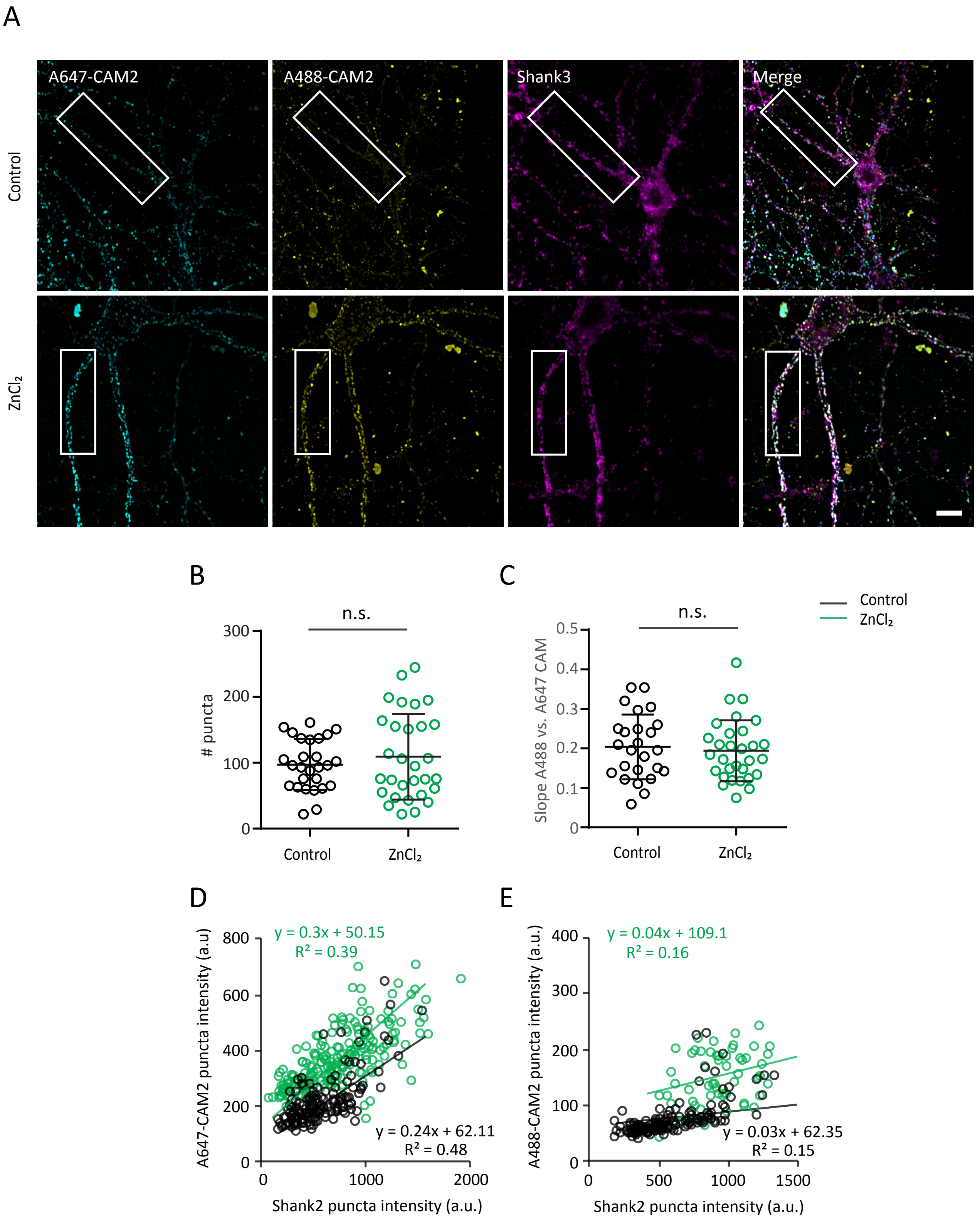

(See next page for Figure legend.)

Supplementary Figure S5 (Cont)

Supplementary Figure S5 | Probing mechanisms of zinc-dependent AMPAR trafficking with CAM2. (A) Hippocampal neurons were labeled with A647-CAM2 (left), then treated with control ($MgCl_2$, top) or $ZnCl_2$ (bottom) conditions before being labeled with A488-CAM2 (second panel from the left) and finally fixed and stained for Shank2 (third panel from the left) or Shank3 (not shown). White boxes mark dendrites shown in **Figure 9**. Scale bar: 20 µm. **(B)** Summary graph showing comparison of the total number of puncta from control and $ZnCl_2$ neurons. [Mann-Whitney test, N = 27 (control) and 30 (ZnCl2) cells from 6-8 coverslips from 4 culture preps; n.s. $p \geq 0.05$]. **(C)** Summary graph showing comparison of the slopes of linear regressions of puncta intensity between A647-CAM2 and A488-CAM2 signal from control and $ZnCl_2$ conditions. [Mann-Whitney test, N = 25 (control) and 30 ($ZnCl_2$) cells from 6-8 coverslips from 4 culture preps; n.s. $p \geq 0.05$]. **(D-E)** Example of fluorescence intensities of individual colocalized CAM2 puncta (A647 or A488) plotted as a function of the corresponding colocalized Shank2 puncta intensities for two neurons. Comparisons were made between sister cultures. Linear regression is shown as a solid line for each condition (black = control; green = zinc).

Supplementary Figure S6

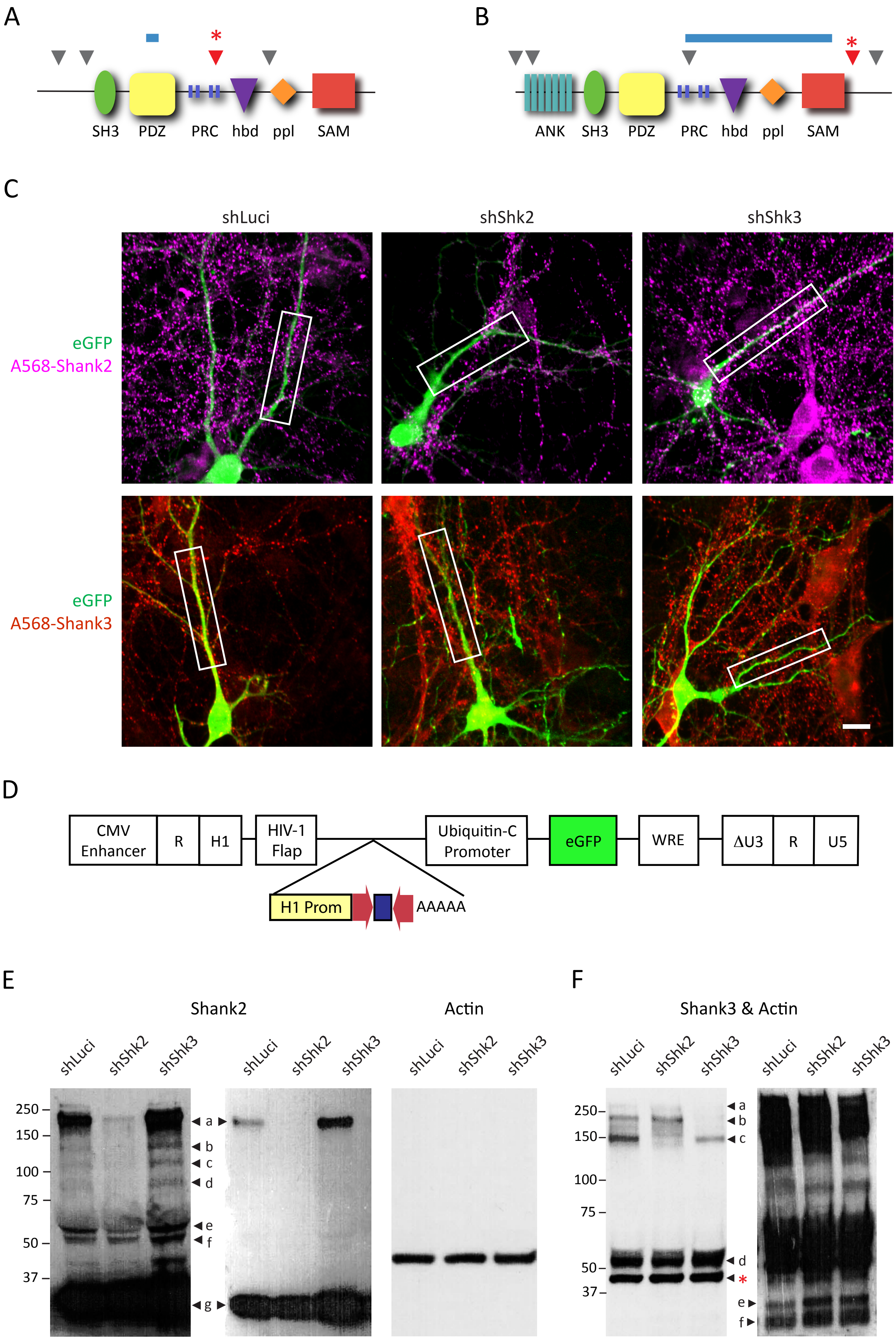

(See next page for Figure legend.)

Supplementary Figure S6 (Cont)

Supplementary Figure S6 | shRNA-mediated knockdown of Shank2 and Shank3. (A-B) Domain structures of Shank2 **(A)** and Shank3 **(B)** including the ankyrin domains (ANK), Src homology 3 (SH3), PSD-95-discs large-zona occludens-1 (PDZ), proline-rich region (PRC), homer binding domain (hbd), the cortactin binding motif (ppl), and a sterile alpha motif (SAM) domain at the C-terminal end. shRNA target sites are marked by gray or red arrow heads. Target sites of shRNA chosen for further experiments are marked by red arrowheads and stars. Peptide regions recognized by Shank2 **(A)** or Shank3 **(B)** antibodies are marked by blue bars. **(C)** Transfected hippocampal neurons expressing eGFP (green) with shLuci (left), shShk2 (middle), shShk3 (right) and immunostained for Shank2 (magenta) or Shank3 (red) after 14 DIV. White boxes indicate dendrites shown in Figure 10. Scale bar: 10 µm. **(D)** Lentiviral bicistronic construct map indicating the shRNA was expressed by H1 promoter and eGFP was expressed by Ubiquitin-C promoter. **(E-F)** Western blots from examples of different exposures shown in **Figure 10**.

Supplementary Figure S7

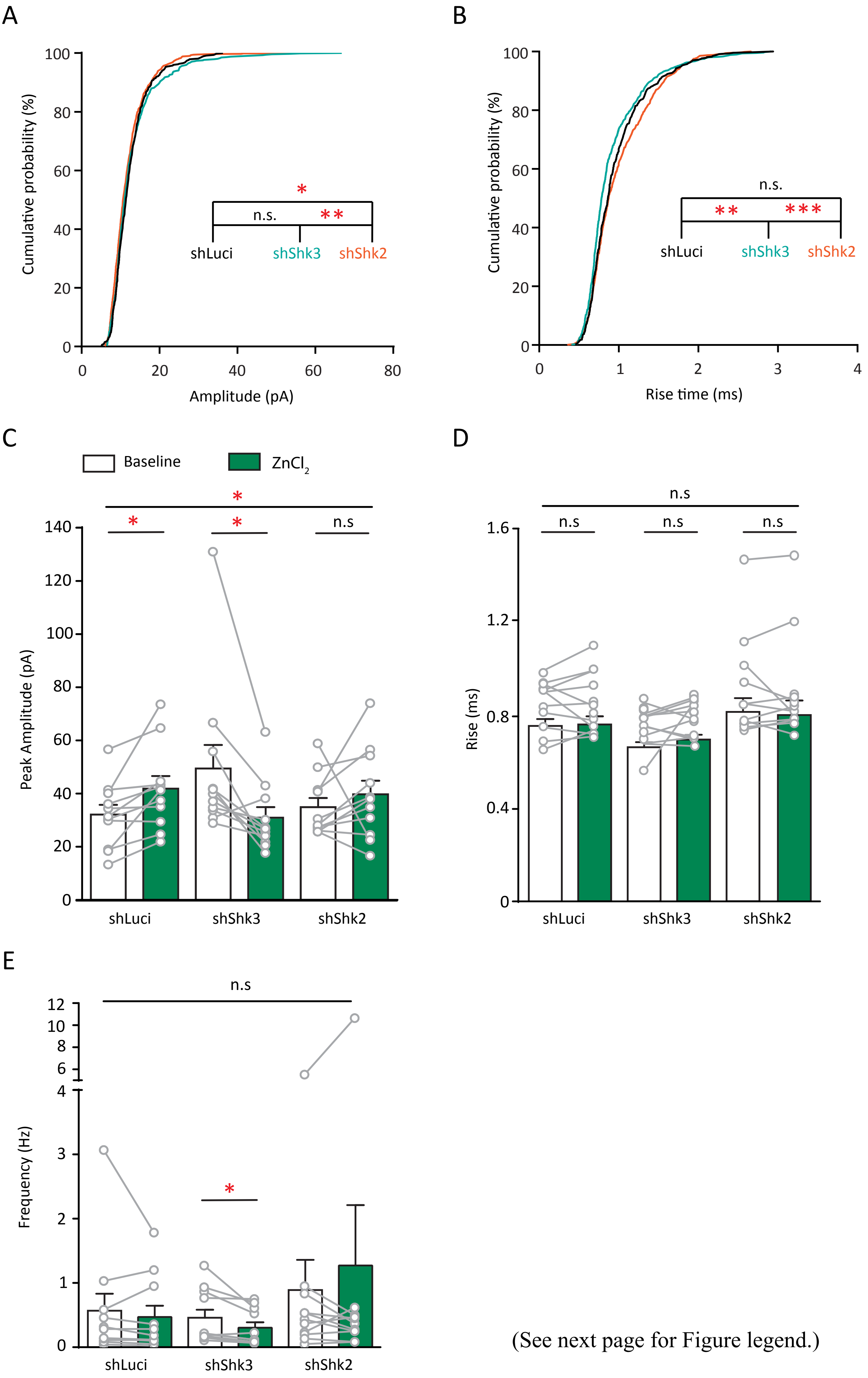

(See next page for Figure legend.)

Supplementary FIGURE S7 (Cont)

Supplementary Figure S7 | Shank knockdown alters AMPAR function and suppresses their zinc-dependent changes. (A-B) Cumulative histogram of amplitude **(A)** and rise time **(B)** from all isolated events of shLuci (black), shShk3 (blue) or shShk2 (orange) neurons. (Kruskal-Wallis one-way ANOVA followed by Dunn's correction post-hoc multiple comparisons, N = 400 – 600 events from 11 neurons from 5-7 culture preps per condition; n.s. $p \geq 0.05$, * $p < 0.05$, ** $p < 0.01$, *** $p < 0.001$). **(C-E)** Per-cell-basis pairwise comparisons of peak amplitude **(C)**, rise time **(D)** and frequency **(E)** between baseline and $ZnCl_2$ conditions of shLuci, shShk3 or shShk2 neurons (mean ± SEM, two-way ANOVA; n.s. $p \geq 0.05$, * $p < 0.05$. Sidak correction multiple post-hoc comparisons, N = 10-11 cells from 5-7 culture preps per condition; n.s. $p \geq 0.05$, * $p < 0.05$).

Supplementary Table 1: Characterization of shRNA targeting Shank2. Sequences shRNA specifically targeting Shank2 are provided in the table below. Knockdown efficiency was examined by transfecting neurons with pZoff construct expressing the shRNA and eGFP, immunolabeling them with Shank2 specific antibodies, and then evaluating the synaptic level of each protein. From + to +++++ : increasing knockdown efficiency. **(*)** indicated the shRNA selected for further experiments in this study.

Target protein	Domain	Sequence (5'-3')	Nucleotide region	Species	Knockdown Efficiency	References
**Shank2** (NCBI Reference Sequence: NM_133441.1)	**5' UTR**	TCGCCTGCTCCATGTTAAC	35-53	Rat	+	Grabrucker et al., 2011
	**Pre-SH3**	TGCCTTCACCAAGAAGGAA	409-427	Rat	+	Grabrucker et al., 2011; Schmeisser et al., 2012
	**Proline Rich**	GGATAAACCGGAAGAGATA	1564-1582	Rat, Ms	+++++ (*)	Berkel et al., 2012
	**Proline Rich**	GGAATTGAGCAAAGAGATT	4399-4417	Rat	++++	Berkel et al., 2012

Supplementary Table 2: Characterization of shRNA targeting Shank3. Sequences shRNA specifically targeting Shank3 are provided in the table below. Knockdown efficiency was examined by transfecting neurons with pZoff construct expressing the shRNA and eGFP, immunolabeling them with Shank3 specific antibodies, and then evaluating the synaptic level of each protein. From + to +++++ : increasing knockdown efficiency. **(*)** indicated the shRNA selected for further experiments in this study.

**Target protein**	**Domain**	**Sequence (5'-3')**	**Nucleotide region**	**Species**	**Knockdown Efficiency**	**References**
**Shank3**<br>(NCBI Reference Sequence: NM_021676.1)	**Pre-Ankyrin**	CGGCAAGTTCCTGGATGAA	425-443	Rat	+	N/A (custom-designed)
	**Ankyrin**	GGTTCTTCGCAATGGCGGT	734-752	Rat	+	Arons et al., 2012; Roussignol, 2005
	**Proline Rich**	GGAAGTCACCAGAGGACAA	3812-3830	Rat, Ms	+++	Bidinosti et al., 2016; Verpelli et al., 2011
	**3' UTR**	GTTTGGAGTCTGGACTAAG	6853-6871	Rat, Ms	++++ (*)	Bidinosti et al., 2016
	**3' UTR**	CACGTCACTCACAAGTTTC	6879-6897	Rat, Ms	+++	Bidinosti et al., 2016
